# Runx2 transcriptome of prostate cancer cells: insights into invasiveness and bone metastasis

**DOI:** 10.1186/1476-4598-9-258

**Published:** 2010-09-23

**Authors:** Sanjeev K Baniwal, Omar Khalid, Yankel Gabet, Ruchir R Shah, Daniel J Purcell, Deepak Mav, Alice E Kohn-Gabet, Yunfan Shi, Gerhard A Coetzee, Baruch Frenkel

**Affiliations:** 1 Department of Biochemistry & Molecular Biology, University of Southern California, Los Angeles, USA; 2 Department of Orthopaedic Surgery, University of Southern California, Los Angeles, USA; 3 Department of Urology, University of Southern California, Los Angeles, USA; 4 Institute for Genetic Medicine, Keck School of Medicine at the University of Southern California, Los Angeles, USA; 5 Norris Cancer Center, University of Southern California, Los Angeles, USA; 6 Keck School of Medicine at the University of Southern California, Los Angeles, USA; 7 SRA International, 2605 Meridian Parkway, Durham, USA; 8 Sciome LLC, 2 Davis Drive, Research Triangle Park, USA

## Abstract

**Background:**

Prostate cancer (PCa) cells preferentially metastasize to bone at least in part by acquiring osteomimetic properties. Runx2, an osteoblast master transcription factor, is aberrantly expressed in PCa cells, and promotes their metastatic phenotype. The transcriptional programs regulated by Runx2 have been extensively studied during osteoblastogenesis, where it activates or represses target genes in a context-dependent manner. However, little is known about the gene regulatory networks influenced by Runx2 in PCa cells. We therefore investigated genome wide mRNA expression changes in PCa cells in response to Runx2.

**Results:**

We engineered a C4-2B PCa sub-line called C4-2B/Rx2^dox^, in which Doxycycline (Dox) treatment stimulates Runx2 expression from very low to levels observed in other PCa cells. Transcriptome profiling using whole genome expression array followed by *in silico *analysis indicated that Runx2 upregulated a multitude of genes with prominent cancer associated functions. They included secreted factors (CSF2, SDF-1), proteolytic enzymes (MMP9, CST7), cytoskeleton modulators (SDC2, Twinfilin, SH3PXD2A), intracellular signaling molecules (DUSP1, SPHK1, RASD1) and transcription factors (Sox9, SNAI2, SMAD3) functioning in epithelium to mesenchyme transition (EMT), tissue invasion, as well as homing and attachment to bone. Consistent with the gene expression data, induction of Runx2 in C4-2B cells enhanced their invasiveness. It also promoted cellular quiescence by blocking the G1/S phase transition during cell cycle progression. Furthermore, the cell cycle block was reversed as Runx2 levels declined after Dox withdrawal.

**Conclusions:**

The effects of Runx2 in C4-2B/Rx2^dox ^cells, as well as similar observations made by employing LNCaP, 22RV1 and PC3 cells, highlight multiple mechanisms by which Runx2 promotes the metastatic phenotype of PCa cells, including tissue invasion, homing to bone and induction of high bone turnover. Runx2 is therefore an attractive target for the development of novel diagnostic, prognostic and therapeutic approaches to PCa management. Targeting Runx2 may prove more effective than focusing on its individual downstream genes and pathways.

## Introduction

Runx2 together with Runx1 and Runx3 comprise the Runx class of transcription factors, defined by their highly homologous Runt-related DNA-binding domain. As heterodimers with Cbfß, Runx proteins bind to cognate DNA elements with the consensus nucleotide sequence 5'-ACCACA in the promoters/enhancers of their target genes [1]. The three Runx proteins coordinate proliferation and differentiation of various cell types [2]. Runx1 is important for hematopoiesis [3,4]; Runx2 is pivotal in osteogenesis [1,5,6]; and Runx3 is critical for neurogenesis [7], thymopoiesis [4], and maintenance of the gastric epithelium [4,8]. While promoting specific cellular phenotypes, Runx proteins have evolved to inhibit cell proliferation. Runx3 is a bona fide tumor suppressor [9] as down-regulation of its promoter by hypermethylation contributes to the development of gastric cancer [10,11]. Ablation of Runx1 activity leads to leukemia [12] and disruption of Runx2 results in deregulated cell proliferation and immortalization [13-17]. Paradoxically, Runx2 is also implicated in carcinogenesis. In a mouse screen for c-Myc-collaborating oncogenes, MLV-induced leukemia occurred most frequently when the provirus integrated into the Runx2 locus resulting in its ectopic expression [18]. It was suggested that Runx2 initially provides the cells with a survival advantage, and its anti-mitogenic activity is counteracted by the CD2-Myc transgene present in the mouse model used for this screen [2,19]. Therefore, Runx2-mediated tumorigenesis likely requires additional loss of check-point genes such as Trp53 or improper regulation of an oncogene such as c-Myc [19].

Runx2 has been extensively studied in the context of osteoblastogenesis from mesenchymal progenitors, where as a master regulator it stimulates the expression of various bone matrix components such as osteocalcin and bone sialoprotein (BSP) [20]. Runx2^-/- ^mice die soon after birth due to the lack of differentiated osteoblasts and thus a mineralized skeleton [1,5,6]. Runx2 haploinsufficiency in humans causes the rare skeletal disorder *Cleidocranial Dysplasia *[21]. In search for hints to explain the high predilection of prostate and breast cancer to metastasize to bone, investigators have noticed ectopic expression of Runx2 and some of its target genes in biopsies from advanced tumors and their derivative cell lines [22-26]. In a mouse model of PCa, conditional deletion of *Pten *in prostate epithelial cells resulted in the development of tumors with progressive increase in Runx2 expression [27]. Among the osteomimetic properties of prostate and breast cancer cells are expression of the Runx2 target genes MMP9 [28], BSP [29] and VEGFA [30], as well as induction of mineralization [25].

In addition to promoting osteoblast differentiation, Runx2 drives the expression of osteoclastogenic signals, both in osteoblasts [31,32] and in the PC3 bone metastasis-derived PCa cell line [22]. PC3 cells robustly express Runx2 [33], and its silencing decreased their osteoclastogenic property *in vitro *and their growth within the bone microenvironment *in vivo *[22]. Runx2 also promotes metastatic aspects not necessarily related to bone. Invasion of PC3 cells through Matrigel™, a basement membrane-like preparation, decreased after Runx2 silencing [22], and its ectopic expression in mammary epithelial cells increased their proliferation and disrupted their normal acinar organization [34]. An oncogenic role for Runx2 has also been suggested in tumors that do not exhibit high predilection to bone, including pancreatic ductal adenocarcinoma [35] and thyroid papillary carcinoma [36].

Whereas Runx2 is being increasingly recognized as a pro-metastatic factor, little is known about the underlying transcriptional programs. To establish gene regulatory networks downstream of Runx2 in aggressive PCa, we analyzed gene expression in response to Runx2 in the C4-2B PCa cell line. These cells are castration-resistant derivatives of the androgen-dependent LNCaP cells, and serve as a model for the aggressive stage of bone metastatic PCa [37,38]. Although C4-2B cells express Runx2 at levels higher than LNCaP cells [25], these levels are far lower than those observed in PC3 cells or osteoblasts [22]. We therefore engineered a C4-2B sub-line that allowed us to profile gene expression after induction of Runx2 with Doxycycline to levels seen in PC3 cells. Remarkably, the most significant changes were the up-regulation of genes implicated in cancer progression and cellular movement, and the down-regulation of genes involved in cell cycle progression. Consistent with these changes in gene expression, Runx2 enhanced PCa cell invasiveness and inhibited their proliferation.

## Results and Discussion

### Establishment of C4-2B PCa cells with conditional Runx2 expression

**Figure 1:**
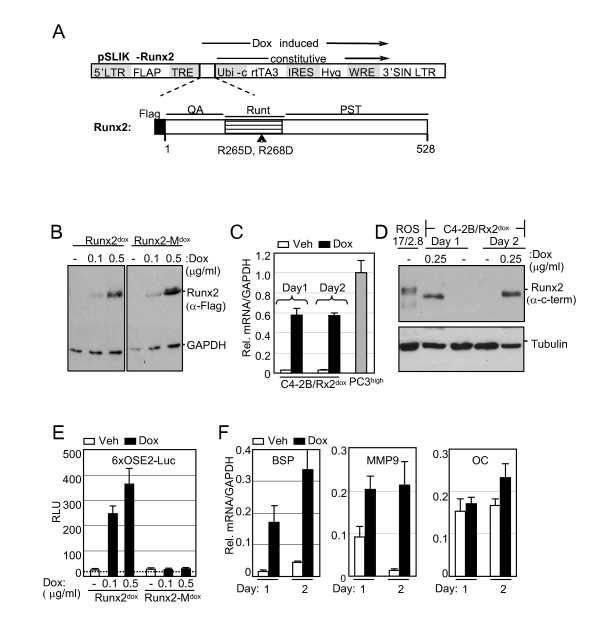
**Establishment of C4-2B/Rx2^dox ^sub-line with conditional Runx2 expression**. A, Schematic diagram of the pSLIK-based lentiviral vector. Initially developed for expression of shRNAs [39] the pSLIK vector was used in the present study to express Runx2. The Hygromycin resistance marker (*Hyg*) and the Dox-dependent activator protein *rtTA3 *are constitutively expressed under the control of the *Ubi-c *promoter. Upon treatment with Dox, rtTA binds to its tetracycline responsive elements (*TRE*) and drives expression of the inserted cDNA (Flag-Runx2). The Runx2 block diagram depicts its glutamine/alanine-rich *QA *domain, the DNA-binding *Runt *domain, and the proline/serine/threonine-rich *PST *domain. Arrowhead indicates the position of the *R265D *and *R268D *mutations in Runx2-M, which eliminate Runx2's DNA binding function. B, Whole cell extracts, prepared from C4-2B/Rx2^dox ^and C4-2B/Rx2-M^dox ^cells treated with the indicated concentrations of Dox, were subjected to western blot analysis using anti-Flag antibodies. C, Total RNA was extracted from C4-2B/Rx2^dox ^cells treated with Dox or vehicle, as well as from PC3^high ^cells, and the mRNA levels of Runx2 (and GAPDH as control) were measured by RT-qPCR. D, Whole cell extracts were prepared from C4-2B/Rx2^dox ^cells treated with Dox as indicated and from ROS 17.8/2 osteoblastic cells [41], and subjected to western blot analysis using anti-Runx2 antibodies. The same blot was re-probed with anti-Tubulin antibodies as loading control. E, C4-2B/Rx2^dox ^and C4-2B/Rx2-M^dox ^cells were transiently transfected with the 6XOSE2-luciferase reporter plasmid and subjected to luciferase assay. Dotted line represents the background luciferase activity with no cell extract. F, C4-2B/Rx2^dox ^cells were treated with Dox and levels of the indicated transcripts were measured by RT-qPCR and corrected for that of GAPDH. In Figure 1, bars represent Mean ± SEM (n = 3) from a representative experiment, which was repeated at least three times with similar results. Abbreviations used: Dox, Doxycycline; Veh, vehicle; BSP, Bone Sialoprotein; MMP9, Matrix Metalloprotein-9; OC, Osteocalcin.

To establish a C4-2B cell line that conditionally expresses Runx2, we employed the recently described lentivirus-based pSLIK vector system, which allows tight Doxycycline (Dox)-inducible, RNA PolII-mediated transcription of a gene of interest [39]. C4-2B cells were transduced with Flag-tagged Runx2-encoding lentiviruses (Figure 1A), resulting in the C4-2B/Rx2^dox ^subline. As control, we established the C4-2B/Rx2-M^dox ^subline, where Dox treatment induced expression of the transcriptionally inactive Flag-Runx2-M (Figure 1A) [40]. Western blot analysis with anti-Flag antibodies confirmed roughly equal expression levels of the wild type and mutant Runx2 proteins, which were strictly and dose-dependently regulated by Dox (Figure 1B). RT-qPCR analysis revealed that the Dox treatment increased Runx2 mRNA by ~20-fold compared to its endogenous levels, and that the induced level was comparable to that observed in the PC3^high ^sub-line (Figure 1C). Western analysis using anti-Runx2 antibodies indicated that the level of endogenous Runx2 protein was negligible in untreated C4-2B cells, and that Dox induced expression of the exogenous Runx2 to the levels normally found in osteoblasts (Figure 1D) [41].

The transcriptional activity of Dox-induced Runx2 was initially assessed using luciferase reporter assay (Figure 1E). In the reporter plasmid 6XOSE2-Luc, luciferase expression was controlled by six copies of the osteoblast-specific element 2 (OSE2) from the Runx2-regulated osteocalcin (OC) gene promoter [42]. In the absence of Dox, 6XOSE2-luc activity was indistinguishable from the background luciferase activity observed without any cell extract, suggesting lack of endogenous Runx2 activity (Figure 1E). The luciferase reporter was strongly stimulated by WT but not by the mutant form of Runx2 (Figure 1E). As shown in Figure 1F, Runx2 also stimulated transcription of its endogenous target genes Bone Sialoprotein (BSP) [29] and Matrix Metalloprotease-9 (MMP9) [28]. These genes were not stimulated in the Dox-treated C4-2B/Rx2-M^dox ^cells (data not shown). Interestingly, Runx2 did not significantly enhance the expression of OC (Figure 1F) and Alkaline Phophatase (ALP; data not shown), although these genes are strongly stimulated by Runx2 in osteoblasts [1,43]. This observation reflects cell type-dependent Runx2-mediated transcriptional control, and is consistent with the results of Yeung *et al*. [33], who demonstrated that in PC3 cells the OC promoter is responsive to the transcription factors AP-1 and SP1, but not Runx2. To identify Runx2-regulated genes and pathways in advanced PCa cells in an unbiased manner, we subjected C4-2B/Rx2^dox ^cells to global gene expression profiling.

### Runx2-regulated global gene expression and *in silico *assessment of associated pathways

C4-2B/Rx2^dox ^cells were subjected to microarray gene expression analysis after one- and two- days of treatment with either Dox or vehicle in biological quadruplicates (a total of 16 samples). Of 24,526 probes represented in the microarray, 532 genes showed ≥2-fold increased expression and 378 genes showed ≥2-fold decreased expression with high statistical significance (*p *< 0.008) on either day of treatment (see additional file 1). RT-qPCR analysis of 50 representative genes conformed to the microarray data (Table 1 and additional file 2).

**Table 1:** Genes most responsive to Runx2 in C4-2B cells

*Gene ID*	*Symbol*	*Gene Name*	*Anova*	*Fold change*			*Description of cancer related or other major function*	*Reference*
			*p value*	I	II	q		
***Transcription regulators***								
7069	THRSP	Thyroid hormone responsive	3.5E-06	7	5		Transcription of enzymes involved in tumor lipid metabolism	
29947	DNMT3L	DNA (cytosine-5-)-methyltransferase 3-like	5.5E-08	5	6	> 50	DNA methylation, nucleic acid metabolic process	
4091	SMAD6	SMAD family member 6	8.1E-10	5	4		Cell communication, TGF-beta receptor signaling pathway	
6591	SNAI2	Snail homolog 2 (Drosophila)	8.8E-07	4	12	6	Promotes EMT by repressing E-cadherin transcription	[44]
6662	SOX9	SRY (sex determining region Y)-box 9	4.4E-07	4	6	17	Promotes chondrocyte differentiation, cell migration, and EMT	[110]
7025	NR2F1	Nuclear receptor subfamily 2,	3.5E-07	3	14	2	Ligand-dependent receptor, regulation of transcription termination	
678	ZFP36L2	Zinc finger protein 36, C3H type-like 2	4.1E-09	3	10		Regulation of RNA metabolic process	
4088	SMAD3	SMAD family member 3	6.3E-10	3	7	25	Signal transducer downstream of TGF-β receptors	
283248	RCOR2	REST corepressor 2	2.9E-06	3	7		Negative regulation of transcription	
10217	CTDSPL	Small CTD phosphatase 3	1.9E-09	-2	-3		Phosphatase, dephosphorylates Rb, suppresses cellular growth	
1870	E2F2	E2F transcription factor 2	8.2E-10	-2	-5	-4	Transcription activator, regulation of cell cycle progression, proliferation	
90390	MED30	Mediator complex subunit 30	< 1.0E-12	-2	-3		Transcription activator, Androgen receptor signaling pathway	
4609	MYC	V-myc myelocytomatosis viral oncogene	2.9E-07	-2	-3	-2	Positive regulation of cell proliferation, apoptosis	[111,112]
29893	PSMC3IP	PSMC3 interacting protein	3.5E-08	-2	-4		Gene expression from RNA PolII	
4602	MYB	Viral oncogene homolog (avian)	1.6E-08	-3	-5	-3	G1/S transition of mitotic cell cycle	
4824	NKX3-1	NK3 homeobox 1	3.7E-08	-3	-3		Transcription repressor, loss induces prostate tumorigenesis	
***Intracellular signaling, cell cycle and proliferation***								
51655	RASD1	RAS, dexamethasone-induced 1	6.8E-12	26	19	> 50	Ras related, suppresses cell proliferation, inhibits tumor growth	[92]
6446	SGK	Serum/glucocorticoid regulated kinase 1	< 1.0E-12	19	45		Cellular Na^+ ^ion homeostasis, cell survival	
57007	CXCR7	Chemokine receptor 7	9.2E-11	8	12	30	Receptor for SDF-1, GPCR, enhanced integrin activation and tumor growth	[113]
1843	DUSP1	Dual specificity phosphatase 1	9.1E-13	8	8	10	Regulator of cell cycle, response to stress	[114]
5580	PRKCD	Protein kinase C, delta	2.0E-11	6	5	12	Serine-Threonine Kinase, cell proliferation, cell contraction	
55890	GPRC5C	G protein-coupled receptor, family C	1.3E-09	5	5	25	GPCR, cell communication	
10461	MERTK	C-mer proto-oncogene tyrosine kinase	6.2E-10	4	6	39	Protein amino acid phosphorylation	
5920	RARRES3	Retinoic acid receptor responder 3	4.3E-08	3	8	40	Negative regulation of cell proliferation	
1903	EDG3	Sphingosine-1-phosphate receptor 3	4.8E-10	3	7		GPCR, enhances tumor growth, cell migration, and angiogenesis	[77-79,115,116]
8877	SPHK1	Sphingosine kinase 1	7.5E-09	3	12	36	Kinase for the production of S1P, enhances tumor growth, cell migration, and angiogenesis, PCa cell survival against chemotherapy, osteoclast chemotaxis	[77-79,115,116]
7049	TGFBR3	Transforming growth factor, β receptor III	7.1E-09	3	4	12	Negative regulation of TGFβ receptor signaling pathway	
4921	DDR2	Discoidin domain receptor tyrosine kinase 2	2.9E-05	2	5		Collagen binding, cell proliferation	
1017	CDK2	Cyclin-dependent kinase 2	1.3E-03	-2	-3	-4	Kinase, mitotic cell cycle, cell proliferation	
55038	CDCA4	Cell division cycle associated 4	7.2E-08	-2	-4	-4	Repressor of E2F-dependent transcriptional activation and cell proliferation	
5997	RGS2	Regulator of G-protein signaling 2, 24 kDa	9.0E-09	-3	-3		Cell cycle, GPCR signaling pathway, cell communication	
25805	BAMBI	BMP and activin membrane-bound inhibitor homolog (Xenopus laevis)	9.7E-10	-2	-3		TGF-β signaling pathway	
***Cellular movement, adhesion, invasion, cytoskeleton remodeling***								
89797	NAV2	Neuron navigator 2	6.7E-09	9	10	29	Scaffold protein, actin cytoskeleton remodeling, nervous system development	[53]
27111	SDCBP2	Syndecan binding protein 2	2.2E-03	6	15		Binds SDC2, regulates cell adhesion and communication with ECM molecules	[84,85]
11344	PTK9L	Twinfilin	9.0E-10	6	5	11	Actin-binding protein, cytoskeleton organization	[57]
83715	ESPN	Espin	2.1E-08	5	8	> 50	Actin-binding-bundling protein, actin cytoskeleton dynamics, cell locomotion	[54,117,118]
51474	LIMA1	Eplin, LIM domain and actin binding 1	3.0E-10	4	5	15	Cytoskeleton-associated protein, regulation of actin filament depolymerization	[56]
8829	NRP1	Neuropilin 1	4.1E-08	4	11	40	TM, coreceptor for VEGF, collagen binding, cell adhesion, invasion	[119]
4582	MUC1	Mucin 1, cell surface associated	1.1E-09	4	4		TM, cell adhesion, invasion	[120]
4131	MAP1B	Microtubule-associated protein 1B	7.1E-09	3	9	18	Trans-membrane glycoprotein, cytoskeleton organization	[55,121]
9644	SH3PXD2A	SH3 and PX domains 2A	1.4E-09	3	7		Adaptor protein, ECM degradation by actin based invadopedia formation	[122]
6275	S100A4	S100 calcium binding protein A4	2.6E-08	2	24	> 50	EF-hand calcium-binding protein, promotes cell motility, tumor metastasis	[49]
6383	SDC2	Syndecan 2	3.1E-06	2	5	32	TM, bind signaling factors, cell adhesion, angiogenesis, ECM degradation	[84,85]
1013	CDH15	Cadherin 15, type 1, M-cadherin	6.9E-08	2	5	21	TM, Ca^2+^dependent intercellular adhesion, skeletal muscle development	[123]
***Secreted factors***								
1437	CSF2	Colony stimulating factor 2/GM-CSF	2.3E-09	12	17	> 50	Cytokine, osteolytic bone metastasis	[72]
5225	PGC	Progastricsin (pepsinogen C)	3.9E-12	11	15		Aspartyl proteinase	[47]
5304	PIP	Prolactin-induced protein	< 1.0E-12	10	79	> 50	Aspartyl proteinase, ligand for CD4 receptor T cells	[46]
8857	FCGBP	Fc fragment of IgG binding protein	1.5E-09	5	9		Cell adhesion, cell-cell recognition	
6387	CXCL12	Chemokine ligand 12 (SDF-1)	2.2E-06	5	7	23	Chemokine, stem cell trafficking to bone, migration, metastasis of tumor cells	[58-65]
7422	VEGFA	Vascular endothelial growth factor A	4.5E-10	5	3	4	Growth factor, angiogenesis, cellular morphogenesis during differentiation	[88-91]
1907	EDN2	Endothelin 2	4.3E-07	5	2		Growth factor, vasoactive, G-protein signaling, smooth muscle contraction	[87]
5654	HTRA1	HtrA serine peptidase 1	6.9E-10	3	18		Serine protease, differentially expressed in osteoarthritic cartillage	[124]
3817	KLK2	Kallikrein-related peptidase 2	1.5E-08	4	3		Serine protease, protein metabolic process	
4880	NPPC	Natriuretic peptide precursor C	1.8E-10	4	17	> 50	Peptide, potent natriuretic, diuretic, and vasodilating	
7057	THBS1	Thrombospondin 1	3.0E-10	4	14	> 50	Cell adhesion, heparin-binding, anti-angiogenic	
3934	LCN2	Lipocalin 2	5.1E-08	4	10	5	ECM protein, present ligands to cell surface receptors, promotes EMT	[45]
6462	SHBG	Sex hormone-binding globulin	8.7E-10	4	10		Steroid-binding, signal	
57642	COL20A1	Collagen, type XX, α 1	4.9E-07	4	10	> 50	Adhesion, establishment of localization, transport	
4254	KITLG	KIT ligand	2.3E-08	4	7	41	Hematopoietic growth factor and ligand for the KIT tyrosine kinase receptor	
4884	NPTX1	Neuronal pentraxin I	2.5E-07	3	20		Cell-cell signaling, transport, establishment of localization	
3486	IGFBP3	IGF binding protein 3	2.2E-08	3	7		Regulation of cell growth, communication, apoptosis	
9806	SPOCK2	Sparc/osteonectin, cwcv, kazal-like domains proteoglycan (testican) 2	1.1E-06	3	4		Cell differentiation, ECM organization and biogenesis	
8530	CST7	Cystatin F (leukocystatin)	3.0E-09	2	25		Competitive inhibitors of cysteine proteases, metastasis associated protein	[48]
4885	NPTX2	Neuronal pentraxin II	7.6E-09	2	6		Cell-cell signaling, synaptic transmission	
147381	CBLN2	ADAM MMP with Thbs-1 motif	5.2E-12	-3	-3		Peptidase, cleavage of precerebellin in the nervous system	
6781	STC1	Spondin 2	3.6E-08	-3	-3		ECM protein, integrin ligand, pattern recognition	
7035	TFPI	Tissue factor pathway inhibitor	3.9E-05	-3	-4		Kunitz-type inhibitor of blood coagulation by blocking factor Xa	
9518	GDF15	Growth differentiation factor 15	1.6E-09	-2	-8	-7	Growth factor, TGF-β signaling pathway, cell communication	

Fold changes on day 1 and day 2 are listed in columns I and II respectively, and column q lists fold changes based on RT-qPCR analysis of the day-2 samples. Major gene functions were determined using knowledge databases from OMIM (Online Mendelian Inheritance of Man), IPA™ systems and the indicated referenced literature. Abbreviations: EMT, epithelial to mesenchymal transition; TM, transmembrane; GPCR, G-protein coupled receptor; ECM, extracellular matrix.

**Figure 2:**
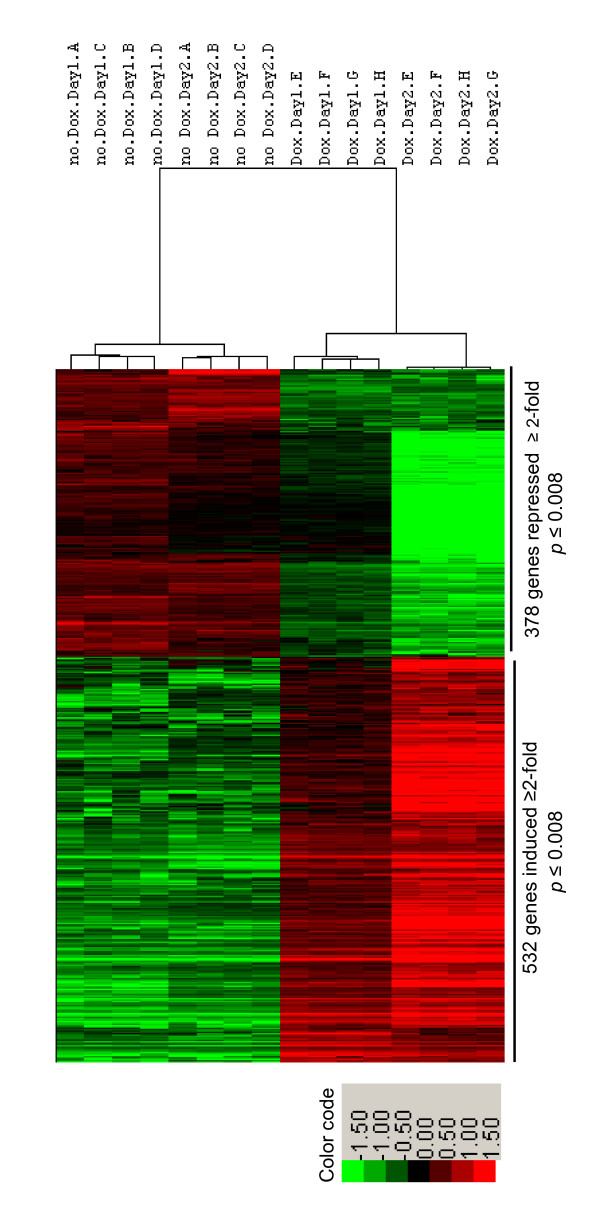
**Unsupervised hierarchical clustering of differentially expressed genes**. Genes that Runx2 up- or down-regulated by ≥2-fold on either day 1 or day 2 (total: 910 genes) with *p *< 0.008 were subjected to pearson's centered correlation matrix. Heatmap represents intensity values relative to the median intensity across all 16 samples per probe after background subtraction and normalization.

An unsupervised hierarchical analysis of these 910 up- and down-regulated genes resulted in a clear separation between the Dox-treated and control samples (Figure 2). The variation among the biological quadruplicates was small, indicating the overall robustness of the methodology utilized. Gene clusters showing changes in expression pattern with respect to time and Dox treatment were clearly discernable. In general, changes observed on day 1 of treatment were maintained or intensified by day 2.

We next employed the Ingenuity Pathway Analysis (IPA™) platform to indentify disease pathways, as well as molecular and cellular functions associated with Runx2-regulated genes. The analysis suggested 'cancer' as the disease most significantly associated with both the up- and the down-regulated gene groups (Table 2). A total of 248 genes, half from each group, had highly significant cancer related function (see additional file 3). Additionally, the up-regulated genes were strongly associated with genetic disorders, inflammatory responses, and gastrointestinal diseases (Table 2A). Among the most significant molecular and cellular functions, cellular movement, cell death, cellular growth, and proliferation were associated with the up-regulated genes, whereas cell cycle, cell death, cellular assembly and DNA replication functions were associated with the down-regulated genes (Table 2B).

**Table 2:** Diseases, Molecular and Cellular Functions associated with genes up-regulated (A) and down-regulated (B) by Runx2

A) ≥ 2 fold up-regulated, total 532 genes	*p-value*	genes
*Diseases and disorders*		
Cancer	2.5e^-07 ^- 3.0e^-03^	124
Genetic disorder	5.4e^-07 ^- 3.0e^-03^	229
Inflammatory response	3.3e^-06 ^- 2.4e^-03^	65
*Molecular and cellular functions*		
Cellular movement	9.7e^-12 ^- 2.5e^-03^	84
Cell death	2.6e^-10 ^- 3.2e^-03^	129
Cellular growth, proliferation	9.3e^-08 ^- 3.2e^-03^	108
**B) ≥ 2 fold down-regulated, total 378 genes**		
*Diseases and disorders*		
Cancer	3.3e^-29 ^- 4.1e^-03^	124
Gastrointestinal disease	3.3e^-29 ^- 3.7e^-03^	72
Genetic disorder	3.3e^-06 ^- 3.4e^-03^	174
*Molecular and cellular functions*		
Cell cycle	7.1e^-35 ^- 4.1e^-03^	102
Cellular assembly, organization	1.7e^-18 ^- 3.4e^-03^	63
DNA replication, recombination, repair	1.7e^-18 ^- 4.1e^-03^	101

IPA™ analysis indicating the association of Runx2-regulated genes to a particular category identifying the indicated disease or function. The ranges of p values indicate the statistical significance with which Runx2-regulated genes associate with the each category.

### Runx2-modulated genes are involved in tumor metastasis

#### Promotion of tissue invasion, metastasis and cytoskeleton dynamics

**Figure 3:**
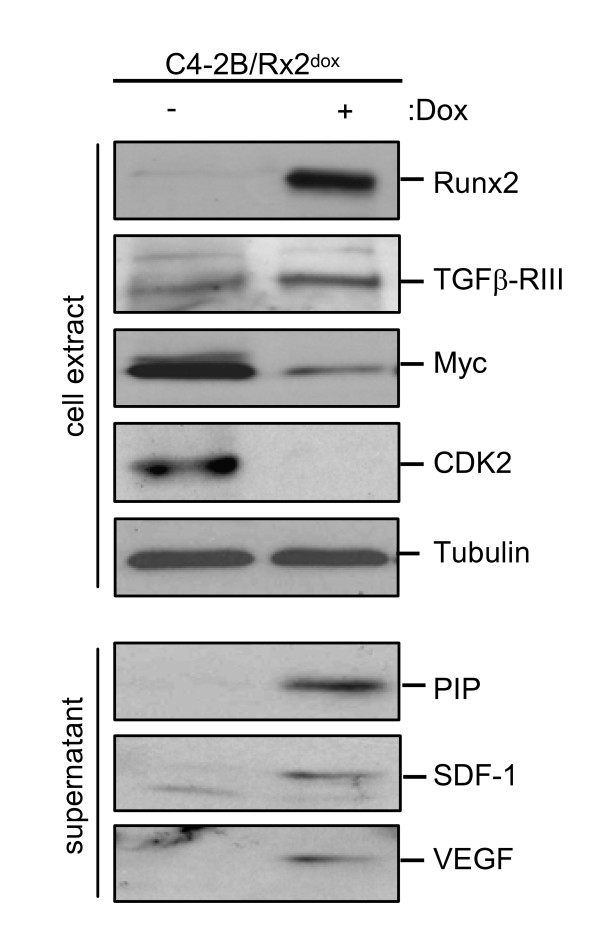
**Runx2-regulated protein expression**. C4-2B/Rx2^dox ^cells were treated with Dox or vehicle control, and proteins extracted as whole cell lysate (*upper panel*) or the supernatant (*bottom panel*) were subjected to western blot analysis using the indicated antibodies.

The major functions reported for the up-regulated genes belonged to cancer progression (Table 2). Importantly, these genes encode transcriptional regulators, cytoskeletal components, signaling molecules and peptidases, which have been implicated in tumor metastasis (Table 1). The transcription factors Sox9 and SNAI2, and the extracellular-matrix (ECM) protein LCN2, all major regulators of epithelial-to-mesenchymal transition (EMT) [44,45], were up-regulated by ~4-fold after one day and by > 6-fold after two days of Runx2 induction, and their upregulation was confirmed by RT-qPCR (Table 1). However, the functional significance of these EMT markers requires further investigation in light of the unexpected increase in E-cadherin mRNA (see additional file 4). Runx2 also enhanced the expression levels of multiple transcripts encoding matrix modifying peptidases (Table 1). These included MMP9, a known Runx2 target in BCa cells [28] and aspartyl proteases with fibronectin degrading activities such as Prolactin Induced Protein (PIP) and Pepsinogen (PGC) [46,47]. The latter two showed a rapid ~10-fold increase within 24 hours (Table 1) and PIP exhibited the highest change (79-fold) in response to Runx2 on day 2 (Table 1 and additional file 2). PIP protein in the C4-2B/Rx2^dox ^culture supernatant was below detectable levels under control conditions, but was readily detected after induction of Runx2 (Figure 3). We also found increased transcript levels for Cystatin 7 (CST7), S100A4 and SMAD3, with a mild ~3-fold increase on day 1, but a robust > 20-fold increase on day 2 (Table 1). These genes function as metastasis promoters [48,49]. Interestingly, S100A4 and SMAD3 physically interact to potentiate cancer cell invasiveness [50].

Runx2 also up-regulated genes involved in cellular movement and cytoskeleton remodeling (Table 1). SH3PXD2A, which was up-regulated by 7-fold on day 2, is a scaffold protein involved in the formation of invadopedia [51,52], which are matrix digesting, actin rich, short lived protrusions observed in osteoclasts and cancer cells [53]. Runx2 up-regulated by > 9-fold the transcripts for Nav2, a scaffold protein crucial for actin cytoskeleton remodeling [53]. Other genes that were up-regulated by > 3-fold, with known roles in actin cytoskeleton dynamics included ESPN, which interacts with the Src-homology 3 (SH3) adaptor proteins to regulate cytoskeletal actin functions [54]; MAP1B, known to maintain cytoskeletal integrity [55]; LIMA1, which cross-links actin monomers [56]; and PTK9L (a.k.a. twinfiln), which sequesters ADP-actin monomers in the cytoplasm and delivers them to sites of rapid actin-filament assembly [57].

#### Metastasis to bone and modification of the bone microenvironment

The expression of SDF-1 and its receptor CXCR7 was enhanced by > 5-fold based on the microarray analysis and by > 20-fold based on the RT-qPCR results (Table 1). Runx2-induced SDF-1 protein was also detectable in the culture supernatant (Figure 3). SDF-1 signaling is critical for homing of hematopoietic cells to the bone marrow space and their survival in this environment [58-65]. Within one day, Runx2 also increased by 10-fold the mRNA for BSP (Figure 1F), whose abundant expression by bone metastatic tumor cells facilitates their attachment to the bone matrix [66-69]. Once settled in the bone microenvironment, the metastatic cells secrete regulatory molecules that stimulate bone turnover [70]. Remarkably, Runx2 enhanced the expression of the osteoclastogenic cytokine CSF2 by > 50-fold within 48 hours (Table 1). This presumably occurred by direct binding of Runx2 to the CSF2 promoter [71]. Runx2-mediated induction of CSF2 in PCa cells likely contributes to the increased bone turnover in bone metastatic sites, similar to the role of this cytokine in breast cancer bone metastasis [72]. CSF2 production by tumor cells may also contribute to accumulation of macrophages, inflammatory T cells, and cytokines [73,74] that exacerbate morbidity and mortality [75]. Two additional Runx2-up-regulated genes associated with osteoclast function are SPHK1, a kinase responsible for the production of sphingosine 1 phosphate (S1P), and S1P receptor 3 (S1P_3_) a.k.a. EDG3 (Table 1). Production of S1P in the bone microenvironment promotes bone resorption by chemotactically attracting osteoclast precursors [76]. The SPHK1/S1P/S1P_3 _axis plays additional roles in cancer progression, including cell growth, migration, angiogenesis, and resistance to chemotherapy [77-79]. Notably, Runx2 was the only gene differentially up-regulated in chemotherapy-resistant versus -sensitive osteosarcoma tumors [80]. Adding to this, Runx2 also repressed the expression of GDF-15, an osteoclastogenesis inhibitor [81] (Table 1). This repression was mild on day 1 (2-fold), but by day 2 GDF-15 was the most repressed gene in response to Runx2 (8-fold; Table 1 and additional file 2). Thus, Runx2-mediated alterations in gene expression may contribute to both the predilection of PCa to bone and the subsequent pathological increase in bone turnover, which further fuels growth of the metastatic tumors.

#### Angiogenesis

Runx2 has been implicated in promoting angiogenesis by stimulating VEGFA expression during bone development [30] as well as during tumorigenesis [82]. In C4-2B/Rx2^dox ^cells, Runx2 increased VEGFA mRNA by 4-fold (Table 1) and the presence of VEGF in the cell culture supernatant was detectable only after Dox treatment (Figure 3). Additionally, our study revealed a 32-fold upregulation of the VEGFA co-receptor Syndecan-2 (SDC2; Table 1). SDC2, which is also a Runx2 target in osteoprogenitor cells [83], is a member of the heparan sulfate proteoglycans family, and is also implicated in cell adhesion and communication [84-86]. Interestingly, VEGFA can functionally synergize with SDF-1 to promote neoangiogenesis *in vivo *[62]. Our microarray analysis also revealed Runx2-mediated induction of the angiogenic EDN-2 gene (Table 1). Endothelins and VEGFA are secreted by PCa cells to stimulate angiogenesis as well as differentiation of neighboring osteoblasts in the bone microenvironment [72,87-91].

### Runx2 increases the invasion potential of C4-2B cells *in vitro*

**Figure 4:**
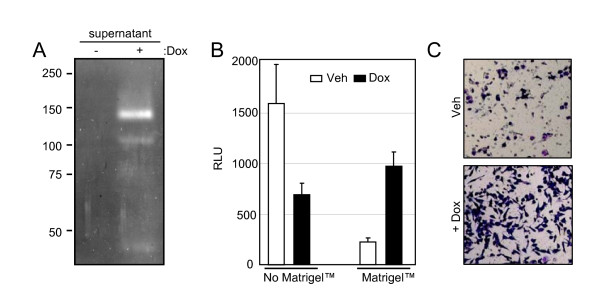
**Runx2 enhances the invasiveness of C4-2B cells**. A) Zymography of supernatants from C4-2B/Rx2^dox ^cells treated with either Dox or vehicle. Negatively-stained bands represent the activity of gelatin-degrading proteases. B) C4-2B/Rx2^dox^/Luc cells were incubated in the top chambers (inserts) of the Matrigel™ invasion system. Cell passage through inserts with and without Matrigel™ represent invasion and migration, respectively. Identical number of cells was seeded in the indicated inserts for 24 hours and the cells that appeared on the bottom side (outside) of the inserts were solubilized in lysis buffer and subjected to luciferase assay. C) The invasion of cells through the Matrigel™ membrane was assessed by staining with Diff-Quick™ solution.

Because Runx2 enhanced the expression of multiple extracellular enzymes involved in ECM degradation (Table 1), we initially tested by in-gel zymography the presence of proteases in the supernatant of Dox-treated C4-2B/Rx2^dox ^cultures. The results demonstrated that Runx2 induced several gelatin-degrading proteins, in particular one with a molecular weight of ~140 kDa, the identity of which remains to be determined (Figure 4A). We further investigated whether Runx2 stimulates invasion of C4-2B/Rx2^dox ^cells through Matrigel™, a tissue basement membrane-like preparation containing laminin, type IV collagen, heparan sulfate proteoglycans and entactin. For convenient and accurate assessment of cells that successfully invade through the Matrigel™ membrane, we transduced the C4-2B/Rx2^dox ^cells with a lentivirus constitutively expressing luciferase. Parallel trans-wells that do not contain Matrigel™ were employed as migration controls. Cells were incubated in the respective chambers in the presence or absence of Dox, and the relative migration or invasion capacity was assessed. Runx2 expression led to a 2.3-fold decrease in cell migration, but a 4.3-fold increase in invasion through Matrigel™, i.e., a ~10-fold increase in invasiveness after adjustment for the reduced cell migration (Figure 4B). The increased invasiveness was further confirmed in an independent experiment by histological staining (Figure 4C). In parallel experiments, expression of Runx2-M in C4-2B cells showed no significant effects on either migration or invasion (data not shown). Thus, expression of transcriptionally active Runx2 is sufficient to enhance the tissue invasion potential of C4-2B cells.

### Runx2 induces cellular quiescence by reversibly inhibiting G1/S cell cycle transition

**Figure 5:**
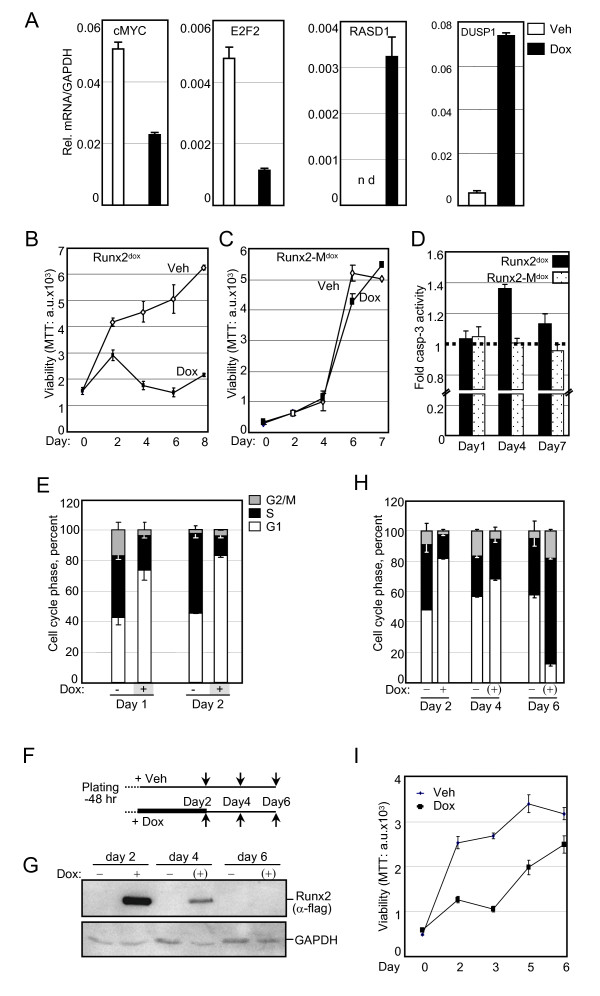
**Runx2 inhibits the G1/S phase transition of the cell cycle**. A, RT-qPCR analysis of the indicated genes in C4-2B/Rx2^dox ^cells treated with Dox (filled bars) or vehicle (open bars) for 48 hours. B and C, MTT-based cell proliferation assays of C4-2B/Rx2^dox ^and C4-2B/Rx2-M^dox ^cells treated with Dox or vehicle as depicted for the indicated time periods. D, Relative apoptosis based on caspase 3 activity in whole cell extracts prepared from C4-2B/Rx2^dox ^and C4-2B/Rx2-M^dox ^cells after treatment with Dox or vehicle for the indicated time periods. E, FACS-based cell cycle analysis of propidium iodide-stained C4-2B/Rx2^dox ^cells treated with Dox for the indicated time periods. F-I, Schematic description of Dox treatment and its subsequent withdrawal from the cell cultures (F). Samples were harvested at the indicated times (arrows) and subjected to analysis of Runx2 levels by western blotting (G), cell cycle profiling by FACS analysis (H), and cell proliferation by MTT assays (I). Abbreviations: RASD1, RAS, Dexamethasone-induced 1; DUSP, Dual Specificity Phosphatase.

The IPA™ analysis of Runx2 down-regulated genes indicated their strong association with cell cycle and proliferation-related functions (Table 2B). Many of these down-regulated genes, as well as several up-regulated genes, shed light on the well-established anti-proliferative activity of Runx2 [2,15-17,19]. Most striking was the > 19- and > 8-fold up-regulation of RASD1 and DUSP1, respectively (Table 1). RASD1 belongs to the Ras superfamily of G-proteins, and its expression in breast cancer suppressed cell growth [92]. DUSP1, a.k.a. MAP kinase phosphatase 1 (MKP1), is a dual specificity (Thr/Tyr) protein phophatase with anti-proliferative properties [93]. Among the most important cell cycle regulatory genes inhibited by Runx2 was c-Myc, with a ~3-fold decrease at the mRNA level (Table 1) and a corresponding significant decrease at the protein level (Figure 3). In line with the down-regulation of c-Myc, the mRNA encoding its cell cycle promoting targets E2F2 and CDK2 were also down-regulated (Table 1). CDK2 protein was decreased below detectability (Figure 3). To further characterize effects of Runx2 on PCa cell proliferation, we first validated by RT-qPCR the changes in the transcript levels of RASD1, DUSP1, c-Myc and E2F2 in the day-2 samples (Figure 5A). Next, we tested the effect of Runx2 on C4-2B cell proliferation by performing MTT assays every 48 hours after Dox-mediated Runx2 induction. Runx2 significantly restrained cell proliferation (Figure 5B). By contrast, the transcriptionally-inactive Runx2-M did not affect proliferation (Figure 5C). Thus, Runx2 restrains PCa cell proliferation *via *its transcriptional activation property.

To delineate the anti-proliferative effect of Runx2, we tested its influence on apoptosis and cell cycle progression. Apoptosis was measured using succinyl-AMC, a fluorogenic Caspase-3 substrate. Expression of Runx2, but not Runx2-M, resulted in a transient 40% increase in apoptosis on day 4 (Figure 5D), but this could not account for the dramatic inhibition of cell proliferation (Figure 5B). Instead, fluorescence activated cell-sorting (FACS) analysis revealed a 2.2-fold decrease in the fraction of cells in the S/G2/M phases of the cell cycle within 24 hours of Dox treatment, and this effect persisted on day 2 (Figure 5E). Notably, the cell cycle inhibition preceded any change in apoptosis, indicating that Runx2 restrained C4-2B cell proliferation by inhibiting the G1/S phase transition of the cell cycle.

The prometastatic but anti-mitogenic properties of Runx2 in PCa cells suggest that it may initially facilitate metastasis, after which it must be degraded or antagonized (e.g. by co-repressors) to allow cell proliferation and tumor growth. Therefore, we examined if the anti-mitogenic effect of Runx2 was reversible by withdrawing Dox from cultures after 48 hours of treatment (Figure 5F). Dox withdrawal led to substantial clearance of Runx2 within two days, and undetectable Runx2 levels after four days (Figure 5G). This resulted in resumption of cell cycle progression (Figure 5H) and cell proliferation (Figure 5I). Thus, the Runx2-regulated gene networks induce reversible cellular quiescence by blocking the G1/S phase transition of the cell cycle.

### Generality and Network modeling of Runx2-regulated genes with cancer-related functions

**Figure 6:**
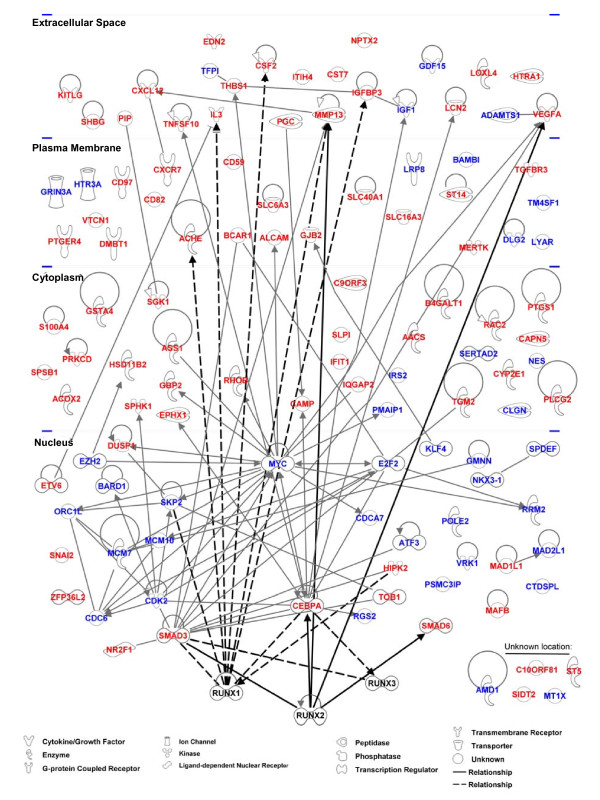
**Runx2-regulated cancer-related gene network**. The 119 cancer-related genes showing a significant ≥2-fold response to Runx2 on day 1 were subjected to the pathway analysis tool from Ingenuity Systems (IPA™). Direct relationships with Runx2 are shown as thick lines and interactions with its paralogs Runx1 and Runx3 are shown as dashed lines. Red and blue fonts mark up- and down-regulated genes, respectively.

Our study is the first to provide genome wide analysis of Runx2-regulated genes in PCa cells. Although we focused on the bone metastasis-derived C4-2B cells, similar responses to Runx2 were observed in the parental lymph node-derived LNCaP cells [38] (see additional file 5) as well as in the unrelated bone metastatic 22RV1 PCa cells (see additional file 6) [94]. Furthermore, in PC3^high ^and PC3^low ^cells with high and low levels of Runx2, respectively, the expression of six randomly selected Runx2 up-regulated genes from the present study correlated with Runx2 expression (see additional file 7). Together, these results suggest that our observations are relevant to various stages of PCa progression. Among the 910 genes that Runx2 up- or down-regulated by ≥2 fold, IPA™ identified 248 genes related to cancer with high statistical significance (*p *< 0.003; Table 2 and additional file 3). The IPA™ analysis, as well as survey of literature on gene expression profiling in osteoblasts and fibroblasts, further suggested that the Runx2-regulated gene network in PCa (Figure 6) bears little resemblance to its targets in mesenchymal cells [43,83,95-97]. In fact, only five of the cancer-related genes in our study have been previously reported as Runx2 targets (MMP13, CEBPA, VEGF, SMAD3, and SMAD6) and only six others are Runx1 and/or Runx3 targets (SKP2, CSF2, IL3, ACHE, IGFBP3, and HIPK2; Figure 6). The remaining 234 genes are therefore novel Runx2-regulated genes related to cancer in general and to metastasis in particular. The extensive Runx2-regulated cancer-related gene network highlights Runx2 as a viable target for the diagnosis, prognosis and treatment of PCa.

## Conclusions

Runx2, traditionally known for its master regulatory roles in the chondro-osteoblast lineage, is emerging as a prometastatic transcription factor [22,29,34,98-101]. The Runx2 transcriptome in C4-2B cells documents gene networks that control multiple aspects of metastasis. Potentially contributing to local invasion and dissemination are the genes known to function in EMT, motility and ECM degradation. Additionally, the prometastatic function of Runx2 likely involves its target genes SDF-1, CXCR7 and BSP, which promote homing and attachment to bone. We also discovered Runx2 targets such as CSF2 and SPHK1, osteoclast activators that likely contribute to the most important alteration that occurs in the bone microenvironment in response to PCa metastasis, namely enhanced bone turnover. During this process, bone matrix components such as TGFß, BMPs and calcium ions are released and further fuel tumor growth and bone microenvironment modifications [102-105]. The regulation of SPHK1 by Runx2 probably potentiates additional aspects of the cancer phenotype, including angiogenesis (a function assisted by the Runx2 targets VEGFA and EDN2) and drug resistance [77,79].

The anti-mitogenic activity of Runx2 is consistent with the slow growth of PCa tumors, and may contribute to drug resistance. We imagine that future anti-Runx2 drugs will be administered along with traditional chemotherapy to eliminate cells that regain proliferative capacity. Interestingly, Runx2 physically and functionally interacts with the receptors for androgens and estrogens [99,101]. Since these receptor proteins are targeted by many drugs for prostate and breast cancer, it is important to investigate their effects on Runx2-regulated transcription. Moreover, development of selective estrogen and androgen receptor modulators may benefit from consideration of their effects on Runx2 and expression of its target genes reported in the present study.

## Methods

### Cell culture reagents and antibodies

C4-2B cells were obtained from ViroMed Laboratories (Minneapolis, MN). LNCaP and 22RV1 cells were from ATCC (Rockville, MD, USA). PC3 cells were also obtained from ATCC, but propagated for several years in either our laboratory (PC3^low^) or that of USC's Dr. Pradip Roy-Burman (PC3^high^). The cells were maintained in RPMI-1640 medium supplemented with 10% Tet System Approved FBS from Clontech, CA, USA. Hygromycin B was purchased from Invitrogen, Carlsbad, CA, USA and added to the growth medium at 50 μg/ml. Doxycycline from Calibiochem, La Jolla, CA, USA was used at 0.25 ug/ml unless otherwise stated, and an equal volume of distilled water was used as vehicle control. Mouse ANTI-FLAG^® ^M2 monoclonal antibody for was purchased from Sigma, St Louis, MO, USA. Mouse anti-Runx2 was from Invitrogen, Carlsbad, CA, USA. The anti-PIP antibody (ab 62363) was purchased from abcam Inc., Cambridge, MA; and anti-GAPDH (V-18), anti-TGFß-RIII (Sc 28975), and anti-SDF-1 (Sc-28876) antibodies were purchased from Santa Cruz Biotechnology Inc, Santa Cruz, CA, USA. The mouse monoclonal anti-Myc and anti-VEGFA antibodies were developed and kindly provided by USC's Dr. Prakash S. Gill and Dr. Young Hong, respectively. The mouse monoclonal anti-Tubulin antibody, developed by Dr. Charles Walsh, was obtained from the Developmental Studies Hybridoma Bank under the auspices of the NICHD and The University of Iowa, Department of Biological Sciences, Iowa City, USA.

### Plasmid construction

The cDNA encoding mouse Runx2 (MASN isoform, type-2), which is 97% identical to human type-2 Runx2 [1], was amplified using pcDNA3.0-Runx2 as template and the Flag epitope was inserted during the PCR amplification (see primers in additional file 8). The Flag-Runx2 cDNA was initially cloned into the SpeI/MfeI-digested lentiviral entry vector pEN_TmiRc3 (ATCC^® ^catalog: MBA-248), and the resulting plasmid was recombined using Gateway^® ^LR Clonase^® ^II enzyme mix (Invitrogen, Carlsbad, CA, USA) with the pSLIK (single lentivector for inducible knockdown) destination vector carrying a hygromycin resistance gene (ATCC^® ^catalog: MBA-237). The entry and destination vectors were kindly provided by USC's Dr. Elizabeth Lowler (Childrens Hospital Los Angeles). The DNA-binding mutant Runx2-M was constructed by site directed mutagenesis of two arginine residues at positions 265 and 268 (GRSGRGK) known to contact DNA in the crystal structure [40]. Lentiviral plasmid for constitutive Luciferase expression pCCL-c-MNDU3c-Luc-PGK-eGFP was kindly provided by USC's Dr. Michael Kahn at the Zilkha Neurogenetic Institute.

### Lentivirus production and infection

For packaging, the lentiviral expression plasmids were cotransfected by the calcium chloride method into HEK293T cells along with helper plasmids pMD.G1 and pCMVR8.91[106,107]. Culture media containing viral particles were harvested after 48-72 hours and used for transduction of C4-2B cells in the presence of 8 μg/ml Polybrene (Millipore Corp., MA, USA). After infection with the lentiviruses, the transduced cells were selected with 50 μg/ml of Hygromycin.

### Transient transfection and Luciferase assays

Transient transfection and Luciferase assays were performed essentially as described by Khalid et al. [99]. Briefly, 25,000 cells were plated in a 24-well plate 48 hours prior to transfection using Invitrogen's Lipofectamine™ LTX reagent according to manufacturer's instructions. Cells were harvested and subjected to luciferase assay using Luciferase Assay system from Promega, Madison, WI, USA.

### High throughput gene expression measurement and analysis

Gene expression profiling was performed using the BeadChip™ platform (Illumina) and chip reference 8, version 3 for humans, which contains 24,526 gene probes and 664 negative control probes. Details of the raw data processing and analysis are provided in the additional file 9. Briefly, after background correction, the normalized expression intensities for all probes were subjected to a two-way analysis of variance (ANOVA) and the resulting *p*-values were adjusted for multiple testing using the Benjimini-Hochberg method [108]. The differentially expressed probes were further investigated using the Ingenuity Pathways Analysis package (IPA™; http://www.ingenuity.com) to identify biological functions and disease categories that are significantly enriched among the differentially expressed genes. Right-tailed Fisher's exact test as implemented in the IPA software was used to calculate a *p*-value for the probability of each network to be enriched for Runx2-regulated genes due to chance alone. The microarray data has been deposited to the GEO database with the accession code GSE24261.

### Preparation of conditioned medium and gelatin zymography

C4-2B/Rx2^dox ^cells were cultured in 10 cm culture dishes to 80% confluence, washed 3 times with RPMI-1640 and treated with Dox in 10 mL of RPMI-1640 without FBS for 24 hrs. For zymography the 25 μl of the conditioned media from Dox treated or control cells were analyzed by 8% acrylamide gels containing 0.1% w/v gelatin [109] After electrophoresis, the gel was washed with 2.5% Triton X-100 and incubated in the developing buffer (500 mM Tris-HCl, pH 7.8, 2 M NaCl, 50 mM CaCl_2_) overnight to induce gelatin lysis. Gel was stained by Coomasie blue-250. For western blot analysis of conditioned media, 250 μl of the supernatant was TCA-precipitated and subjected to reducing SDS-PAGE analysis using standard procedures.

### In vitro invasion and migration assays

Invasion through Matrigel™ was assessed by incubating 20,000 luciferase-expressing cells at the top of 24 well BD BioCoat™ Growth Factor Reduced Matrigel™ chamber (BD Biosciences, MA, USA). Migration was assessed using BD BioCoat™ Control Inserts. Cells that migrated or invaded to the bottom compartment were visualized by Diff-Quick™ staining kit (Baxter, MN, USA) or subjected to luciferase assays for quantification. Invasion index was defined as the percentage of cells that invaded through Matrigel™ over those that migrated under the same conditions but without the Matrigel™.

### Proliferation, cell cycle and apoptosis

Cultures on different days were subjected to MTT assay (Sigma, St Louis, MO) to measure viable cells in culture. For cell cycle analysis, 2 × 10^5 ^to 1 × 10^6 ^cells were harvested, washed twice with PBS (1 mL) at room temperature and stored in absolute ethanol (4 mL) for at least 24 hours. Pelleted cells were rehydrated in 5 mL PBS for 15 minutes, followed by staining with 1 mL of a propidium iodide (PI, Calibiochem, La jolla, CA, USA) solution containing 3 μM PI in incubation buffer (100 mM Tris, pH 7.4, 150 mM NaCl, 1 mM CaCl_2_, 0.5 mM MgCl_2_, 0.1% Nonidet^® ^P-40). The cell suspension (1 mL) was subjected to fluorescence-activated cell sorting (FACScaliber, Becton Dickinson, MA, USA) and each cell was assigned to the G1, S, G2 or M phase of the cell cycle based on the PI intensity and using the Multicycle v3.0 software (Phoenix Flow Systems, San Diego, CA, USA). To assess apoptosis, cells were lysed in caspase assay buffer containing 50 mM HEPES (pH 7.5), 100 mM NaCl, 2 mM EDTA, 0.1% CHAPS, 10% sucrose, and 5 mM DTT. Aliquots of crude cell lysate (50 μg protein) were incubated with the caspase-3 substrate Ac-DEVD-AMC (EMD/Calbiochem, La Jolla, CA, USA) at 37°C for 30 min and the caspase-3 activity was quantified by flow *fluorimetry *with excitation at 380 nm and emission at 440 nm using Victor_3_V™ from PerkinElmer, Shelton, CT, USA.

### Western blot analysis

Between 1 × 10^5 ^and 2 × 10^5 ^cells were washed once with PBS and lysed with 200 μL of incubation buffer [100 mM Tris (pH 7.4), 500 mM NaCl, 1 mM CaCl_2_, 0.5 mM MgCl_2_, 0.1% Nonidet^® ^P-40] supplemented with Complete™ protease inhibitor mix (Roche Diagnostics, Indianapolis, IN, USA). Aliquots of 40 μg cell lysate were mixed with an equal volume of Laemmli buffer and proteins were resolved by 10% SDS-PAGE, transferred to Amersham Hybond™-P PVDF (GE Healthcare, Piscataway, NJ, USA) membranes, and visualized using respective antibodies and the Western Lightning™ Plus-ECL kit (PerkinElmer Inc, Waltham, MA, USA) followed by exposure to X-ray film (ISCBioExpress^®^, Kaysville, UT, USA).

### RT-qPCR

Total RNA was isolated using Aurum Total RNA kit (Bio-Rad Laboratories Inc., Hercules, CA, USA) following the manufacturer's recommendations and 1 μg was reverse transcribed using the Superscript III kit (Invitrogen, CA, USA). The cDNA was subjected to real-time PCR amplification using iQ SYBR Green Supermix and a Opticon™2 real time PCR machine from Bio-Rad, Hercules, CA. The sequences of primers for amplification of the cDNA of interest and the control GAPDH are listed in the additional file 8.

GEO accession code for the microarray data: GSE24261

## Competing interests

The authors declare that they have no competing interests.

## Authors' contributions

Conceived and designed the experiments: SKB and BF. Performed the experiments: SKB, OK, YG, AEG, SS and DJP. Bioinformatics analysis: RRS and DM. Analyzed data and wrote the paper: SKB, GAC and BF. All authors have read and approved the final manuscript.

## Supplementary Material

Additional file 1 **ANOVA analysis of the microarray data**. List of all probes with the microarray gene expression data in log scale (base 2) along with the Fold change, P-values and gene annotations. Click here for file

Additional file 2 **Runx2-regulated genes involved in cellular metabolism**. List of genes with functions in cellular metabolism, their fold changes, and known functions. Click here for file

Additional file 3 **Runx2 -regulated genes involved in cancer**. List of 256 genes with established roles in cancer. Click here for file

Additional file 4 **E-Cadherin expression in C4-2B/Rx2^dox ^cells upon Runx2 expression**. Western blot and RT-qPCR analysis of C4-2B/Rx2^dox ^cells in response to Runx2 expression. Click here for file

Additional file 5 **Generation and characterization of LNCaP/Rx2^dox ^cells**. RT-PCR to detect Runx2 transcript in PC3, C4-2B, and LNCaP cells. Proliferation of the LNCaP/Rx2^dox ^cells by using MTT, and RT-qPCR analysis of Runx2-regulated genes. Click here for file

Additional file 6 **Generation and characterization of 22RV1/Rx2^dox ^cells**. Western blot analysis of Dox-induced Runx2, and MTT based proliferation analysis of 22RV1/Rx2^dox ^cells in response to Runx2 expression. Click here for file

Additional file 7 **Expression of PGC, CST7, S100A4, SDF-1, CSF2, and DUSP1 in two PC3 sub-lines with different Runx2 levels**. RT-qPCR analysis using PC3^high ^and PC3^low ^cells to examine the expression of Runx2-regulated genes. Click here for file

Additional file 8 **List of primers used in the present study to PCR-amplify the indicated gene sequences**. Nucleotide sequence of primers. Click here for file

Additional file 9 **Bioinformatics analysis of the microarray data**. Detailed description of microarray quality control and data processing (such as quality control, normalization and box/density plots); and methodology employed for the identification of differentially expressed probes. Click here for file
