# Supplementary material for: Runx2 transcriptome of prostate cancer cells: insights into invasiveness and bone metastasis
Source: Mol Cancer. 2010 Sep 23;9:258. doi: 10.1186/1476-4598-9-258 (PMC2955618; doi:10.1186/1476-4598-9-258)
Supplement: Additional file 2 — Runx2-regulated genes involved in cellular metabolism. List of genes with functions in cellular metabolism, their fold changes, and known functions. [file 1476-4598-9-258-S2.DOC]

| Additional file 2 ***Metabolite transportation, ion homeostasis and other functions*** | | | | | | | | | | | |
| --- | --- | --- | --- | --- | --- | --- | --- | --- | --- | --- | --- |
| *Gene ID* | *Symbol* | *Gene Name* | | | *Anova* | *Fold change* | | | | | *Description of major function* |
|  |  |  | | | *p* value | I | | II | | |  |
| 2348 | FOLR1 | | folate receptor 1 | 2.1E-07 | | | 19 | | 6 | transporter, organic acid metabolic process, establishment of cellular localization | |
| 189 | AGXT | | alanine-glyoxylate aminotransferase | 2.0E-09 | | | 12 | | 17 | enzyme , oxalic acid secretion, establishment of cellular localization | |
| 6531 | SLC6A3 | | solute carrier family 6, member 3 | 2.7E-09 | | | 10 | | 3 | transporter , dopamine uptake, cell-cell signaling | |
| 2938 | GSTA1 | | glutathione S-transferase alpha 1 | 1.7E-11 | | | 9 | | 38 | enzyme, cellular metabolic process | |
| 6523 | SLC5A1 | | solute carrier family 5, member 1 | 2.1E-07 | | | 7 | | 6 | transporter , Na^+^/glucose co-transport, urogenital system development | |
| 80176 | SPSB1 | | splA/ryanodine receptor domain and SOCS box containing 1 | 2.5E-09 | | | 5 | | 6 | cell communication, protein modification process | |
| 6590 | SLPI | | secretory leukocyte peptidase inhibitor | 9.1E-13 | | | 5 | | 7 | acute inflammatory response, urea metabolic process | |
| 2939 | GSTA2 | | glutathione S-transferase alpha 2 | 1.6E-07 | | | 5 | | 14 | enzyme , metabolic process | |
| 5742 | PTGS1 | | prostaglandin-endoperoxide synthase 1 | 5.3E-07 | | | 5 | | 5 | enzyme , prostaglandin biosynthetic process, cellular developmental process | |
| 3709 | ITPR2 | | inositol 1,4,5-triphosphate receptor, type 2 | 1.1E-07 | | | 4 | | 6 | ion channel, calcium ion transport, establishment of localization | |
| 1497 | CTNS | | cystinosis, nephropathic | 1.2E-04 | | | 4 | | 12 | carboxylic acid metabolic process, establishment of localization | |
| 2517 | FUCA1 | | fucosidase, alpha-L- 1, tissue | <1.0E-12 | | | 4 | | 10 | enzyme , carbohydrate metabolic process | |
| 3291 | HSD11B2 | | hydroxysteroid (11-beta) dehydrogenase 2 | 1.0E-09 | | | 4 | | 10 | enzyme , cell-cell signaling, positive regulation of blood pressure | |
| 6337 | SCNN1A | | sodium channel, nonvoltage-gated 1 α | 9.4E-11 | | | 4 | | 4 | transporter , ion channel, monovalent inorganic cation transport | |
| 221357 | GSTA5 | | glutathione S-transferase alpha 5 | 3.2E-07 | | | 3 | | 9 | enzyme , metabolic process | |
| 2634 | GBP2 | | guanylate binding protein 2, interferon-inducible | 1.6E-07 | | | 3 | | 11 | enzyme , immune system process | |
| 6295 | SAG | | S-antigen; retina and pineal gland | 6.1E-05 | | | 3 | | 8 | sensory perception | |
| 342035 | GLDN | | gliomedin | 1.4E-06 | | | 3 | | 27 | ion transport | |
| 1571 | CYP2E1 | | cytochrome P450, family 2, subfamily E1 | 3.5E-08 | | | 3 | | 21 | enzyme , generation of precursor metabolites and energy | |
| 54020 | SLC37A1 | | solute carrier family 37, member 1 | 1.3E-09 | | | 3 | | 5 | intracellular transporter, glycerol-3-phosphate transport, carbohydrate transport | |
| 30061 | SLC40A1 | | solute carrier family 40, member 1 | 1.4E-07 | | | 3 | | 4 | transporter , Proton coupled divalent metal ion | |
| 3632 | INPP5A | | inositol polyphosphate-5-phosphatase | 3.1E-09 | | | 3 | | 4 | enzyme, phosphatase, carbohydrate metabolic process | |
| 2706 | GJB2 | | gap junction protein, beta 2 | 4.2E-09 | | | 3 | | 4 | transporter , cell communication, sensory perception of mechanical stimulus | |
| 4056 | LTC4S | | leukotriene C4 synthase | 2.4E-07 | | | 2 | | 12 | enzyme , organic acid and lipid metabolic process | |
| 445 | ASS | | argininosuccinate synthetase 1 | 1.2E-09 | | | 2 | | 7 | enzyme , urea cycle, response to glucocorticoid stimulus, acute inflammatory response | |
| 820 | CAMP | | cathelicidin antimicrobial peptide | 1.5E-05 | | | 2 | | 8 | carbohydrate metabolic process | |
| 9056 | SLC7A7 | | solute carrier family, member 7 | 2.8E-07 | | | 2 | | 9 | transporter , cationic amino acid transport, carboxylic acid transport | |
| 81035 | COLEC12 | | collectin sub-family member 12 | 1.1E-10 | | | 2 | | 7 | transporter , ion transport, Tm receptor | |
| 2902 | GRIN1 | | glutamate receptor | 6.5E-07 | | | 2 | | 4 | transporter , ion channel, cellular calcium ion homeostasis | |
| 8911 | CACNA1I | | calcium channel | 1.3E-03 | | | 1 | | 6 | transporter , ion channel, tri-valent inorganic cation transport | |
| 7296 | TXNRD1 | | thioredoxin reductase 1 | 2.0E-06 | | | -2 | | -4 | enzyme, response to oxidative stress, homeostatic process | |
| 151126 | ZNF385B | | zinc finger protein 385B | <1.0E-12 | | | -2 | | -5 | other | |
| 55540 | IL17RB | | interleukin 17 receptor B | 4.6E-09 | | | -2 | | -4 | Tm receptor, regulation of cell growth | |
| 55646 | LYAR | | Ly1 antibody reactive homolog (mouse) | 6.5E-08 | | | -2 | | -3 | other, regulation of cell growth, anatomical structure morphogenesis | |
| 6860 | SYT4 | | synaptotagmin IV | 1.1E-07 | | | -2 | | -2 | transporter, cell-cell signaling, regulation of neurotransmitter levels | |
| 79993 | ELOVL7 | | ELOVL family member 7, elongation of long chain fatty acids (yeast) | 2.0E-06 | | | -2 | | -3 | other | |
| 9920 | KBTBD11 | | kelch repeat and BTB (POZ) domain containing 11 | 3.7E-08 | | | -2 | | -3 | Other | |
| Additional file 2 *(continued)* | | | | | | | | | | | |
| *Gene ID* | *Symbol* | | *Gene Name* | *Anova* | | | *Fold change* | | | | *Description of major function* |
|  |  | |  | *p* value | | | I | | II | |  |
| 7035 | TFPI | | tissue factor pathway inhibitor (lipoprotein-associated coagulation inhibitor) | 3.9E-05 | | | -2 | | -3 | | other, response to wounding |
| 8825 | LIN7A | | lin-7 homolog A (C. elegans) | 1.8E-10 | | | -3 | | -5 | | other, vesicle-mediated transport, cell-cell signaling |
| 85414 | SLC45A3 | | solute carrier family 45, member 3 | 3.0E-10 | | | -3 | | -3 | | other, transport, establishment of localization |
| 9510 | ADAMTS1 | | ADAM metallopeptidase with thrombospondin type 1 motif, 1 | 1.7E-08 | | | -3 | | -3 | | peptidase, reproductive developmental process, |
| 10417 | SPON2 | | spondin 2, extracellular matrix protein | 1.9E-08 | | | -3 | | -3 | | other, cell adhesion |
| 6781 | STC1 | | stanniocalcin 1 | 3.6E-08 | | | -3 | | -4 | | kinase, cellular cation and anion homeostasis |
| 7296 | TXNRD1 | | thioredoxin reductase 1 | 2.0E-06 | | | -3 | | -3 | | enzyme, response to reactive oxygen species |
| 262 | AMD1 | | adenosylmethionine decarboxylase 1 | 2.3E-09 | | | -3 | | -2 | | enzyme, nucleobase, nucleoside, nucleotide and nucleic acid metabolism |
| 388394 | RPRML | | reprimo-like | 6.5E-11 | | | -4 | | -8 | | other |
