# Supplementary material for: Runx2 transcriptome of prostate cancer cells: insights into invasiveness and bone metastasis
Source: Mol Cancer. 2010 Sep 23;9:258. doi: 10.1186/1476-4598-9-258 (PMC2955618; doi:10.1186/1476-4598-9-258)
Supplement: Additional file 4 — E-Cadherin expression in C4-2B/Rx2dox cells upon Runx2 expression. Western blot and RT-qPCR analysis of C4-2B/Rx2dox cells in response to Runx2 expression. [file 1476-4598-9-258-S4.PDF]

Additional file 4

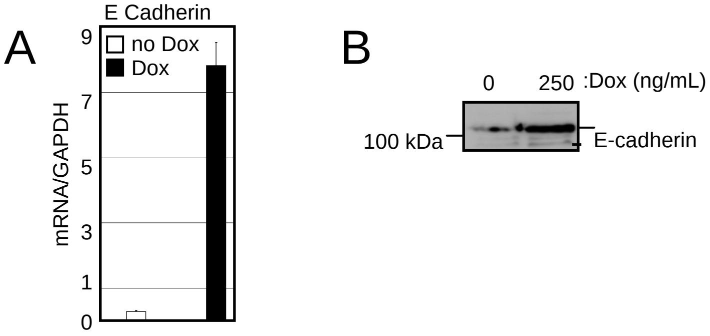

Additional file 4: **E-Cadherin expression in C4-2B/Rx2^{dox} cells upon Runx2 expression**. A) RT-qPCR and C4-2B/Rx2^{dox} cells were treated with Doxycycline (Dox) and E-Cadherin transcript levels were measured by RT-qPCR and corrected for that of GAPDH. B) Whole cell extracts prepared from C4-2B/Rx2^{dox} cells treated with Dox or vehicle control were subjected to western blot analysis using E-Cadherin antibodies.

The increase in E-Cadherin expression is unexpected since we also observed that Runx2 increased expression of SNAI2 (Table1), which is a negative regulator of E-Cadherin expression (Peinado et al., MCB Vol 24, page 306, 2004)
