# Supplementary material for: Runx2 transcriptome of prostate cancer cells: insights into invasiveness and bone metastasis
Source: Mol Cancer. 2010 Sep 23;9:258. doi: 10.1186/1476-4598-9-258 (PMC2955618; doi:10.1186/1476-4598-9-258)
Supplement: Additional file 5 — Generation and characterization of LNCaP/Rx2dox cells. RT-PCR to detect Runx2 transcript in PC3, C4-2B, and LNCaP cells. Proliferation of the LNCaP/Rx2dox cells by using MTT, and RT-qPCR analysis of Runx2-regulated genes. [file 1476-4598-9-258-S5.PDF]

Additional file 5

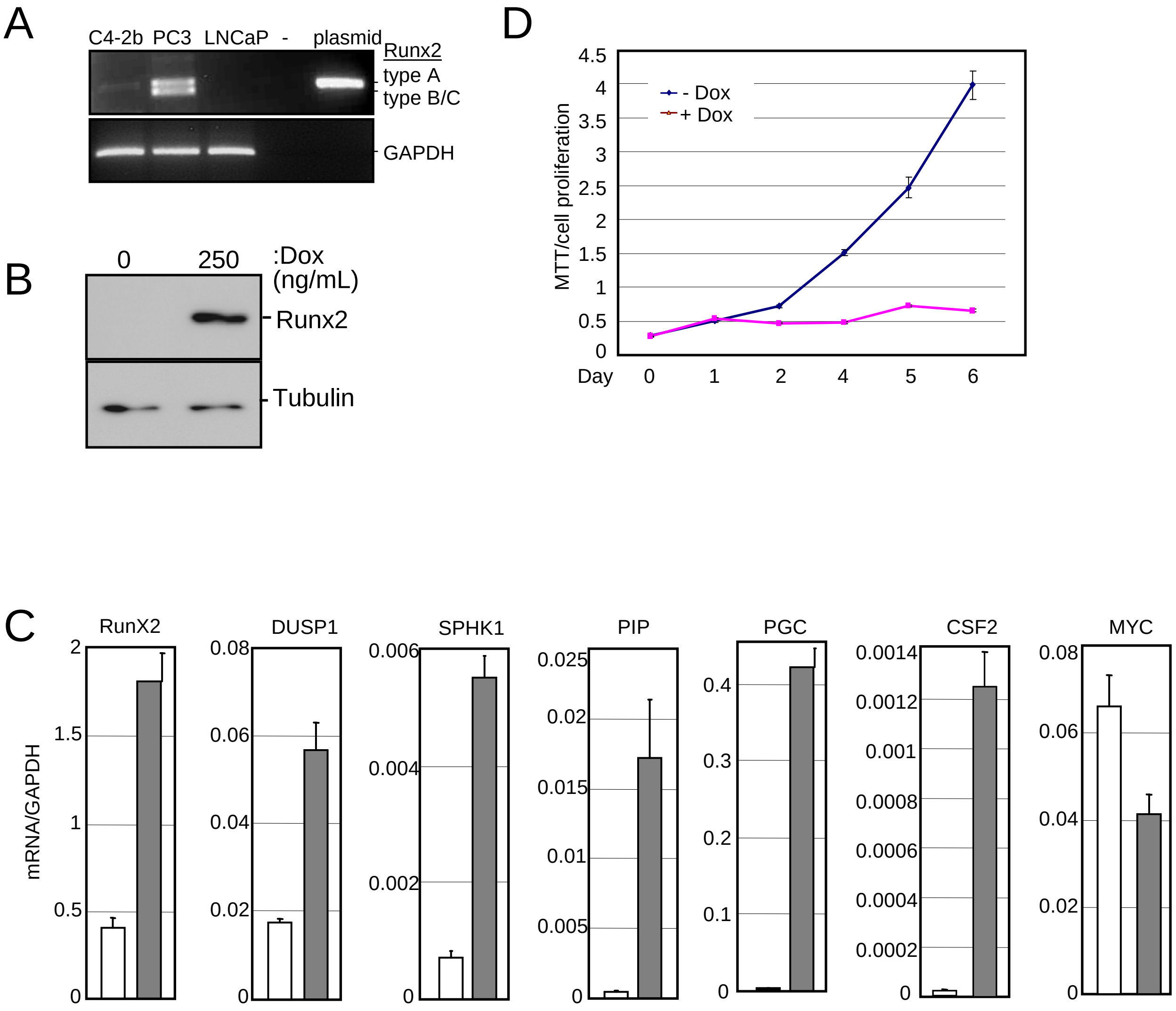

Additional file 5: **Generation and characterization of LNCaP/Rx2^{dox} cells.** A) Agarose gel electrophoresis of PCR amplicons after RT-PCR to assess Runx2 transcript levels in C4-2B, PC3, and LNCaP cell lines. Specific primer pairs recognizing all three isoforms of Runx2 were used. Plasmid control was type-A Runx2 cloned in a pcDNA3.0 vector kindly provided by Dr. Jennifer J. Westendorf at the Department of Orthopaedic Surgery, University of Minnesota, Minneapolis. B) Whole cell extracts prepared from LNCaP/Rx2^{dox} cells treated with Doxycycline (Dox) vehicle or control were subjected to western blot analysis using anti-Flag and anti-Tubulin antibodies. C) LNCaP/Rx2^{dox} cells were treated with Dox and levels of the indicated transcripts were measured by RT-qPCR and corrected for that of GAPDH. Bars represent Mean±SEM (n=3) from a representative experiment, which was repeated at least three times with similar results. D) MTT-based cell proliferation assays of LNCaP/Rx2^{dox} cells treated with Dox or vehicle as depicted for the indicated time periods.
