# Supplementary material for: Runx2 transcriptome of prostate cancer cells: insights into invasiveness and bone metastasis
Source: Mol Cancer. 2010 Sep 23;9:258. doi: 10.1186/1476-4598-9-258 (PMC2955618; doi:10.1186/1476-4598-9-258)
Supplement: Additional file 6 — Generation and characterization of 22RV1/Rx2dox cells. Western blot analysis of Dox-induced Runx2, and MTT based proliferation analysis of 22RV1/Rx2dox cells in response to Runx2 expression. [file 1476-4598-9-258-S6.PDF]

Additional file 6

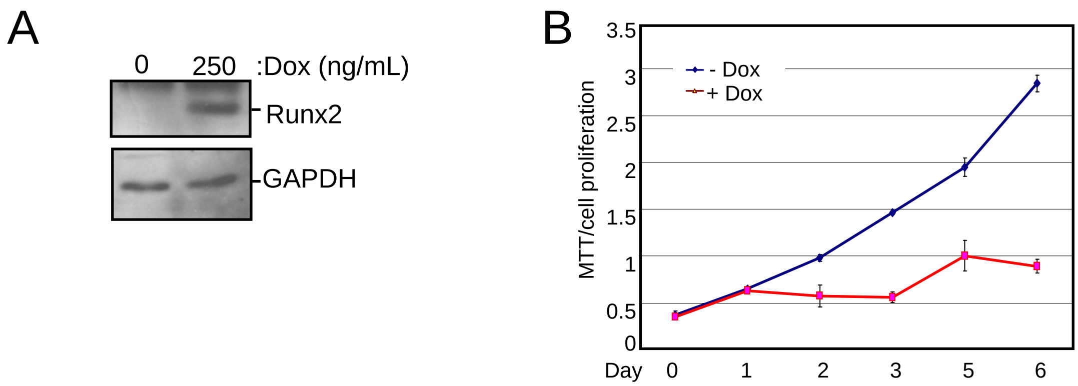

Additional file 6: **Generation and characterization of 22RV1/Rx2^{dox} cells.** A) Western blt analysis using the whole cell extracts prepared from 22RV1/Rx2^{dox} cells treated with Doxycycline (Dox) or vehicle control were subjected to western blot analysis using anti-Flag and anti-GAPDH antibodies. B) MTT-based cell proliferation assays of 22RV1/Rx2^{dox} cells treated with Dox or vehicle as depicted for the indicated time periods.
