# Supplementary material for: Runx2 transcriptome of prostate cancer cells: insights into invasiveness and bone metastasis
Source: Mol Cancer. 2010 Sep 23;9:258. doi: 10.1186/1476-4598-9-258 (PMC2955618; doi:10.1186/1476-4598-9-258)
Supplement: Additional file 7 — Expression of PGC, CST7, S100A4, SDF-1, CSF2, and DUSP1 in two PC3 sub-lines with different Runx2 levels. RT-qPCR analysis using PC3high and PC3low cells to examine the expression of Runx2-regulated genes. [file 1476-4598-9-258-S7.PDF]

Additional file 7

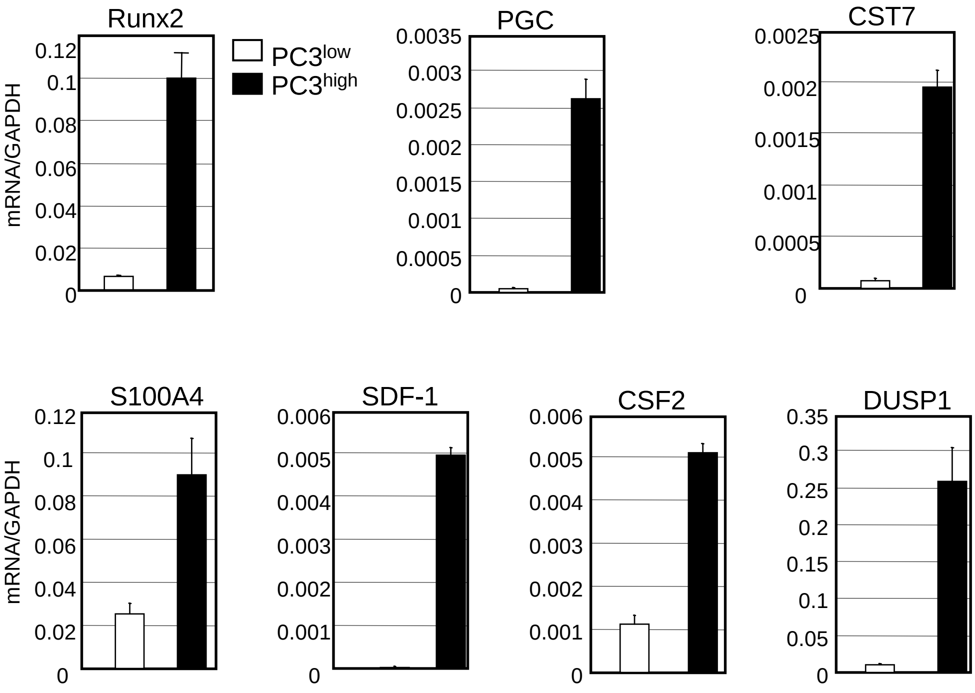

Additional file 7: **Expression of PGC, CST7, S100A4, SDF-1, CSF2, and DUSP1 in two PC3 sub-lines with different Runx2 levels.** Expression of the indicated genes was measured by RT-qPCR in $PC3^{high}$ and $PC3^{low}$ cells (see Methods). Results (Mean±SEM; n=3) were corrected for expression of GAPDH.
