# Supplementary material for: Runx2 transcriptome of prostate cancer cells: insights into invasiveness and bone metastasis
Source: Mol Cancer. 2010 Sep 23;9:258. doi: 10.1186/1476-4598-9-258 (PMC2955618; doi:10.1186/1476-4598-9-258)
Supplement: Additional file 8 — List of primers used in the present study to PCR-amplify the indicated gene sequences. Nucleotide sequence of primers. [file 1476-4598-9-258-S8.DOC]

| **Additional file 8: Primers used for RT-PCR** | | |
| --- | --- | --- |
| **Gene** | **Forward (5’ to 3’)** | **Reverse (5’ to 3’)** |
| Flag-Runx2 | ATGGACTACAAAGACGATGACGACC  TTCATTAGCCTCACAAACAAC | CTCGAGCAATTGTCAATATGGCCGCCAAACAG |
| ALPL | CACCCACGTCGATTGCATCT | TAGCCACGTTGGTGTTGAGC |
| CDCA4 | TTTCTGACCTTTGCCCAGTC | TCAGGTCTCCACCAGGATCT |
| CDH15 | CGAGTTCACCAGGGATGAGTT | GAACACCCTCGTTGGTCTTGG |
| CDK2 | TTTGCTGAGATGGTGACTCG | AAGGGTGGTGGAGGCTAACT |
| COL20A1 | AGGGGTCAAAGGAGAGAAGG | TCTCGTGGAAGGAGTCGAAC |
| CSF-2 | ATGTGAATGCCATCCAGGAG | AGGGCAGTGCTGCTTGTAGT |
| CST7 | TCCCAGGACCTTAACTCACG | GCTTCAAGGTGTGGTTGGTT |
| CXCL12 | ATGAACGCCAAGGTCGTG | CTTTAGCTTCGGGTCAATGC |
| CXCR7 | GGCTATGACACGCACTGCTACA | TGGTTGTGCTGCACGAGACT |
| DNMT3L | CTCTCAAGCTCCGTTTCACC | CGACTCTCGGTCGTAGAAGG |
| DUSP1 | GCCACCATCTGCCTTGCTTAC | CTCGGTCGAGCACAGCCATG |
| E2F2 | TCCTGAGCGAGTCAGAGGAT | TGTCCTCAGTCAGGTGCTTG |
| EDN2 | TGGGTGAACACTCCTGAACA | GAAATGTCCCTCAGCCTTTG |
| ESPN | CAGAGTGCAGGACAAAGACAA | TAGTGCTCGACGAGAAGCCT |
| FOLR1 | TGCCTGCAAACGGCATTTC | CACGTTCAGTACCCGCTCTTT |
| GAPDH | GTCATGGGTGTGAACCATGAGA | GGTCATGAGTCCTTCCACGATAC |
| GDF15 | ACCTGCACCTGCGTATCTCT | CGGACGAAGATTCTGCCAG |
| GMCSF | ATGTGAATGCCATCCAGGAG | AGGGCAGTGCTGCTTGTAGT |
| GPRC5C | GCTGCTGACCAGTGTGTACC | CAGTTCGCTTACCTGAGAGTTC |
| IBSP | TGGATGAAAACGAACAAGGCA | AAACCCACCATTTGGAGAGGT |
| KITLG | CAAAAATCCCCCTGGAGAC | TTGAAACTCTCTCTCTTTCTCTTGC |
| LIMA1 | CACTGAGATTAGGCACAGAGC | ACCGTCCTTGATGTGGGGA |
| LTC4S | GCTGTTCCTCGCCAC | TAGCCCTGGAAGTAGCG |
| MAP1B | TACGTGGTGAGTGGGAATGA | ACTGTGCTGCTGCTTGCTAA |
| MERTK | AGTGTGCAGCGTTCAGACAAT | AACCCAGAAAATGTTGACGGG |
| MMP9 | TTGACAGCGACAAGAAGTGG | GCCATTCACGTCGTCCTTAT |
| MYB | AAGTCTGGAAAGCGTCACTTG | ACATCTGTTCGATTCGGGAGATA |
| MYC | AGAGAAGCTGGCCTCCTACC | CGTCGAGGAGAGCAGAGAAT |
| NAV2 | GCTGCTTCCTTGTGGCTATC | TAACCCCGTCTGTCAACTCC |
| NPPC | GGCACCATGCATCTCTCC | CCTTCTTGTTGGCTCCTTTG |
| NPTX1 | GCCAAGTTGCCTTTTGTCAT | ATCTCGATGTGGGATTCAGC |
| NR2F1 | AGAAGCTCAAGGCGCTACAC | CCTACCAAACGGACGAAGAA |
| NRP1 | ATCACCCAAGTGAAAAATGCGA | GAGGGGCTATCTTTCCACAGA |
| OC | GGCAGCGAGGTAGTGAAGAG | CTGGAGAGGAGCAGAACTGG |
| PGC | ACAGGCACCTCTCTGCTAACT | AGTAGCCGTTGTTACTGAGGAT |
| PIP | GTACGTCCAAATGACGAAGTCAC | CAGCAGCATCATCAGGGCAGATG |
| PRKCD | AGGCCAAGGTGTTGATGTCT | AGGTGGCGATAAACTCATGG |
| PTK9L | CTACCGCCTCGACTCACAGA | ATCCTTCACAGTCCCGAAGAG |
| RANKL | TCAGAAGATGGCACTCACTG | AACATCTCCCACTGGCTGTA |
| RARRES3 | TGGCTCCTCCAGTGTCTTCT | TGCTCACAGTTCCTGCTCAC |
| RASD1 | GTGTTCAGTCTGGACAACCGC | CTGCTCGATCTCGCGCTGGTC |
| RCOR2 | TTGACAAGTACATTGCGATGGC | CTACTGTCCACTCGTCAGGGA |
| RUNX2 | CACGAATGCACTATCCAGCCAC | CGCCAAACAGATTCATCCATTC |
| SDC2 | GCAATCGCTGCGGTACTCT | TTCTTCAATGGAGCTGTTGTCAA |
| SH3PXD2 | ATGATCCTGGAACAGTACGTGG | TCTTAGAGGTGTTGATGTCGGA |
| SHB | GAAGGAGCGACACTTCCAGG | CTGCGGTAGAGATGCGGAG |
| SLC45A3 | GACACTATGATGAAGGCGTTCG | GAGAAGGTGAACCCGGTGAG |
| SMAD3 | GGGCTCCCTCATGTCATCTA | ATTCGGGGATAGGTTTGGAG |
| SNAI2 | ATATTCGGACCCACACATTACCT | GCAAATGCTCTGTTGCAGTGA |
| SOX9 | GAGGAAGTCGGTGAAGAACG | ATCGAAGGTCTCGATGTTGG |
| SPHK1 | TCTGGTGGTCATGTCTGGAG | CACAGCAATAGCGTGCAGTT |
| TGFBR3 | ACCTGTCAGTGCCTCCCAT | GAGCAGGAACACAACAGACTT |
| THBS1 | GGGGGCGTCAATGACAATTTC | GCCAATGTAGTTAGTGCGGAT |
| TSPAN32 | GCCCAGCCTCTTGAGATGC | CCACCGAAGACTACCCGAG |
| E-cadherin | TTCCTCCCAATACATCTCCC | TTGATTTTGTAGTCACCCACC |
| VEGFA | TCTTCAAGCCATCCTGTGTG | CCTCGGCTTGTCACATTTTT |
