# Supplementary material for: Runx2 transcriptome of prostate cancer cells: insights into invasiveness and bone metastasis
Source: Mol Cancer. 2010 Sep 23;9:258. doi: 10.1186/1476-4598-9-258 (PMC2955618; doi:10.1186/1476-4598-9-258)
Supplement: Additional file 9 — Bioinformatics analysis of the microarray data. Detailed description of microarray quality control and data processing (such as quality control, normalization and box/density plots); and methodology employed for the identification of differentially expressed probes. [file 1476-4598-9-258-S9.DOC]

Additional file 9: **Bioinformatics analysis of the microarray data**

*Microarray data processing and identification of differentially expressed genes*:

Four biological replicates each from treatment (Dox) and control (no-Dox) group post day 1 and day 2 of exposure were utilized to prepare total of 16 RNA samples. For the dox treated samples RNA was prepared from the cells after treating them with dox for 24 hrs (day 1) or for 48 hrs (day 2). As control, RNA from side-by-side cultured dox untreated cells was prepared. Resulting RNA samples were hybridized to Human ref 8 version 3 Illumina microarrays. Raw signal intensities were obtained from the BeadStudio output files. Each of the 16 samples included expression data for 24,526 probes and 664 negative control probes.

First, we performed Principal Component Analysis (PCA) on background corrected normalized data for all probes and all samples as implemented in the programming language R [3] to look for general effects in the dataset. We observed that samples clearly separated by their ‘treatment’ type (Dox vs. Control) and separation was greater in the Day 2 samples compared to Day 1 [Figure 2].

To identify probes that show statistically significant differential expression due to treatment and duration of treatment, we used the background corrected and normalized expression intensities for each probe in a two-way analysis of variance (ANOVA) analysis (see methods section for more details). The resulting p-values were adjusted for multiple testing using the Benjimini Hochberg method (False Discovery Rate). Probes were ranked based on their adjusted p-values and fold change. Probes that displayed Fold change of two fold or greater in either direction (up and down regulation), along with p-values less than 0.05 were selected for further analysis. This included 532 probes that showed increased expression (up-regulation) and 378 probes that shown decreased expression (down regulation) in Dox treated group as compared to control group. An unsupervised hierarchical clustering of samples based on the expression profiles of the above list of probes resulted in a clear separation between the Dox and Control samples (Figure SN2).

Data processing was performed in two stages: First, we performed background correction to remove background (noise) from the expression data and in the second stage we normalized the data so to remove across sample variation as described below.

The background correction was performed using the Model-based Background Correction for BeadArrays (MBCB) method [1]. This methodology utilizes negative control probes along with test probes to estimate the unobservable background intensities using the normal exponential deconvolution model similar to the Robust Multi-array Average (RMA) normalization approach [2]. It estimates background intensities and unknown parameters using Markov Chain Monte Carlo simulations.

The between sample variation in the log2-transformed background corrected signal was accounted for using quantile normalization [2] as implemented in bioconductor ([www.bioconductor.org](http://www.bioconductor.org/)). To check for any anomalies and/or outliers in the data, we plotted the expression intensities of each probe in a sample as a density plot [Figure SN3] and box plot [Figure SN4] as implemented in bioconductor. We do not find any outliers or anomalies in the dataset; hence expression data for all probes and all samples was used for further analyses.

*Statistical analysis to identify differentially expressed genes*:

Identification of differentially expressed genes: To identify probes that show statistically significant differential expression due to treatment and duration, we used the background corrected and normalized expression intensities for each probe in a two-way analysis of variance (ANOVA) analysis implemented in the R programming language [3]. This model included two main effects: day effect and treatment effect along with their interaction effect. The false discovery rates (FDR) for each probe were computed from p-values for significance of treatment effect from type-I analysis (also known as variable added in order approach) using Benjimini Hochberg method [4].

FIGURES

Figure SN1


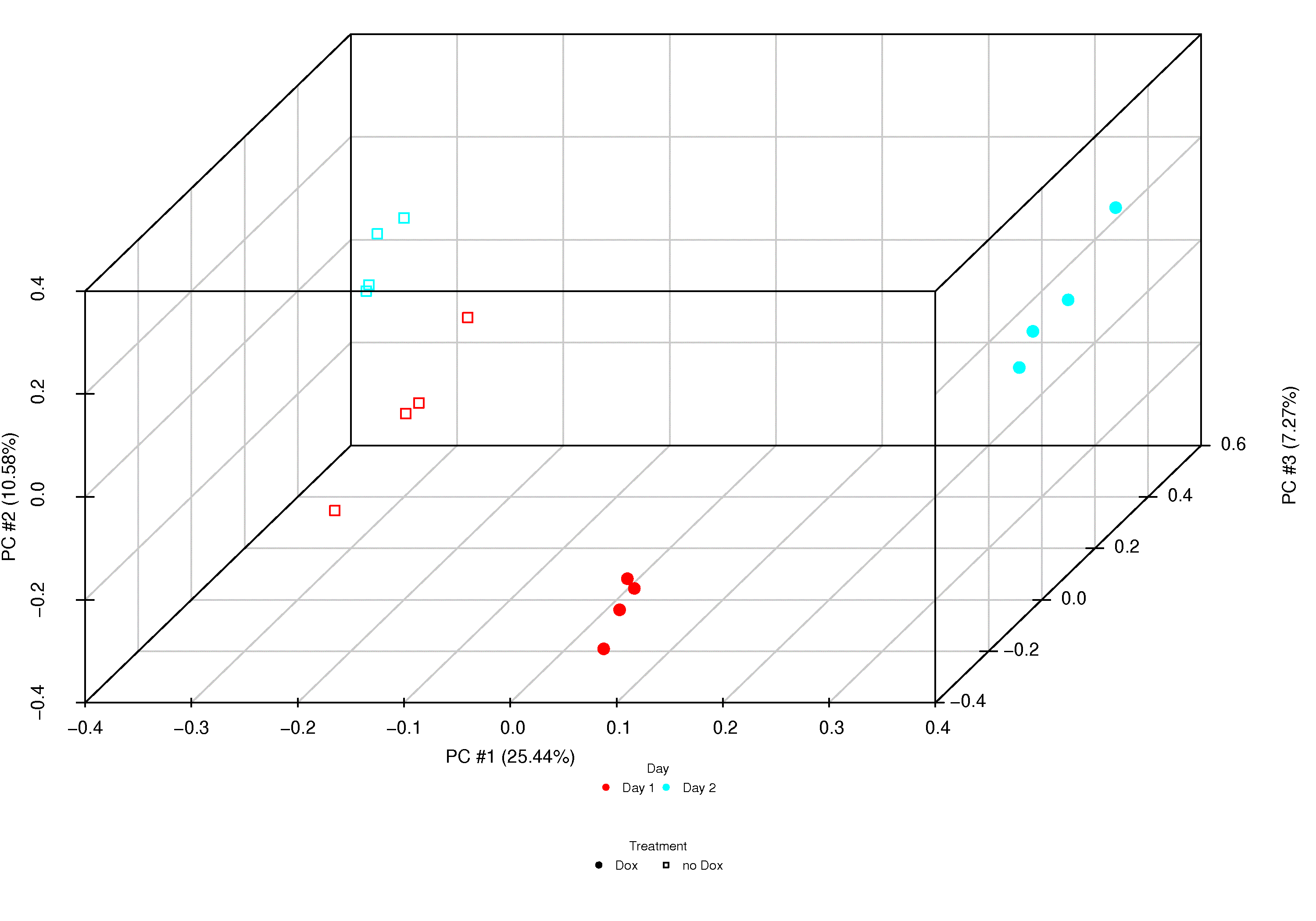


Figure SN2 Legend: Principal component analysis using whole genome gene expression data suggests a clear separation between Dox treated and untreated groups. Samples are further separated on Day 2 compared to Day 1.

Figure SN2

Figure SN3


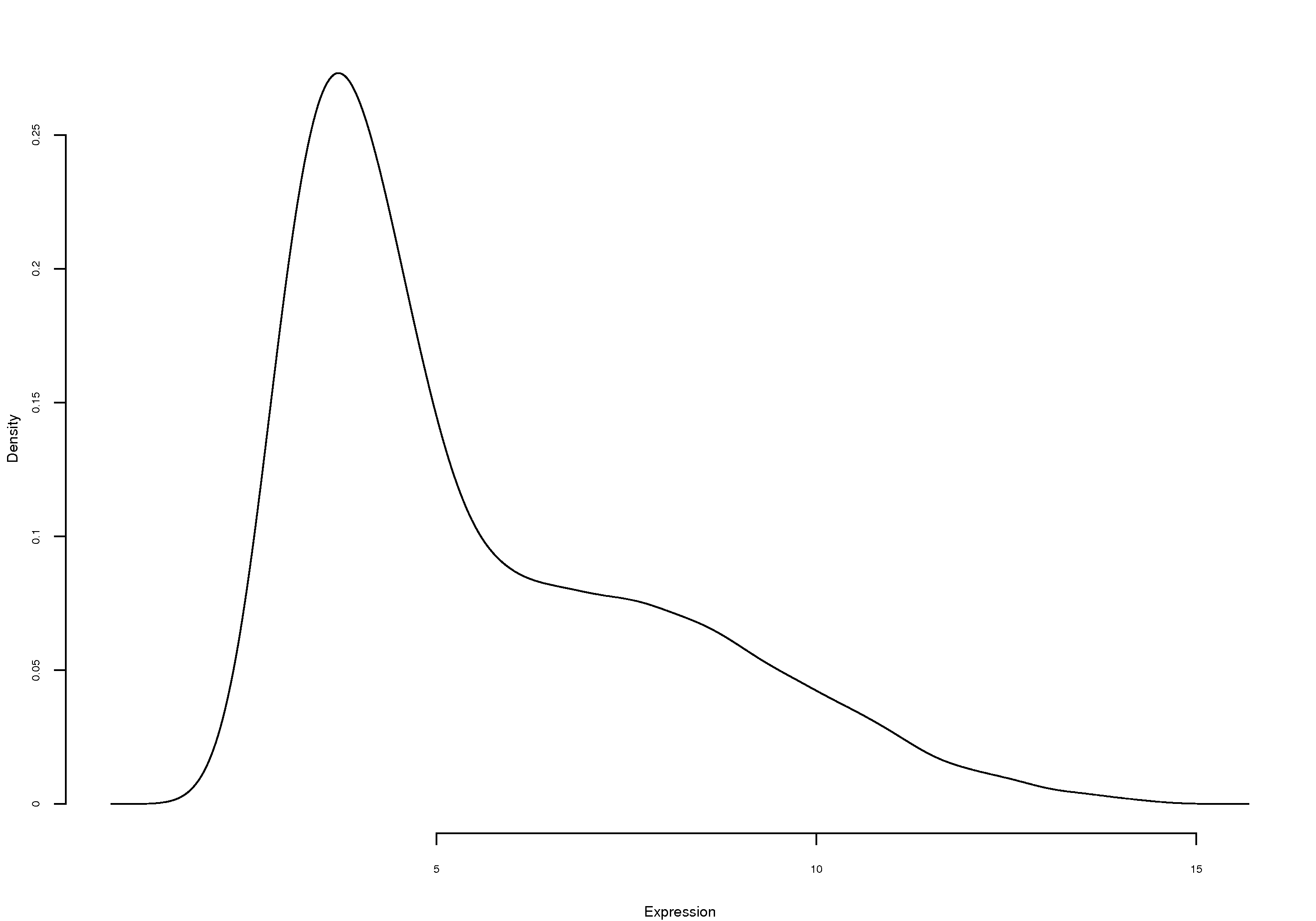


Figure SN3 Legend; Density plot of background corrected normalized intensity values (in log 2 scale).

Figure SN4


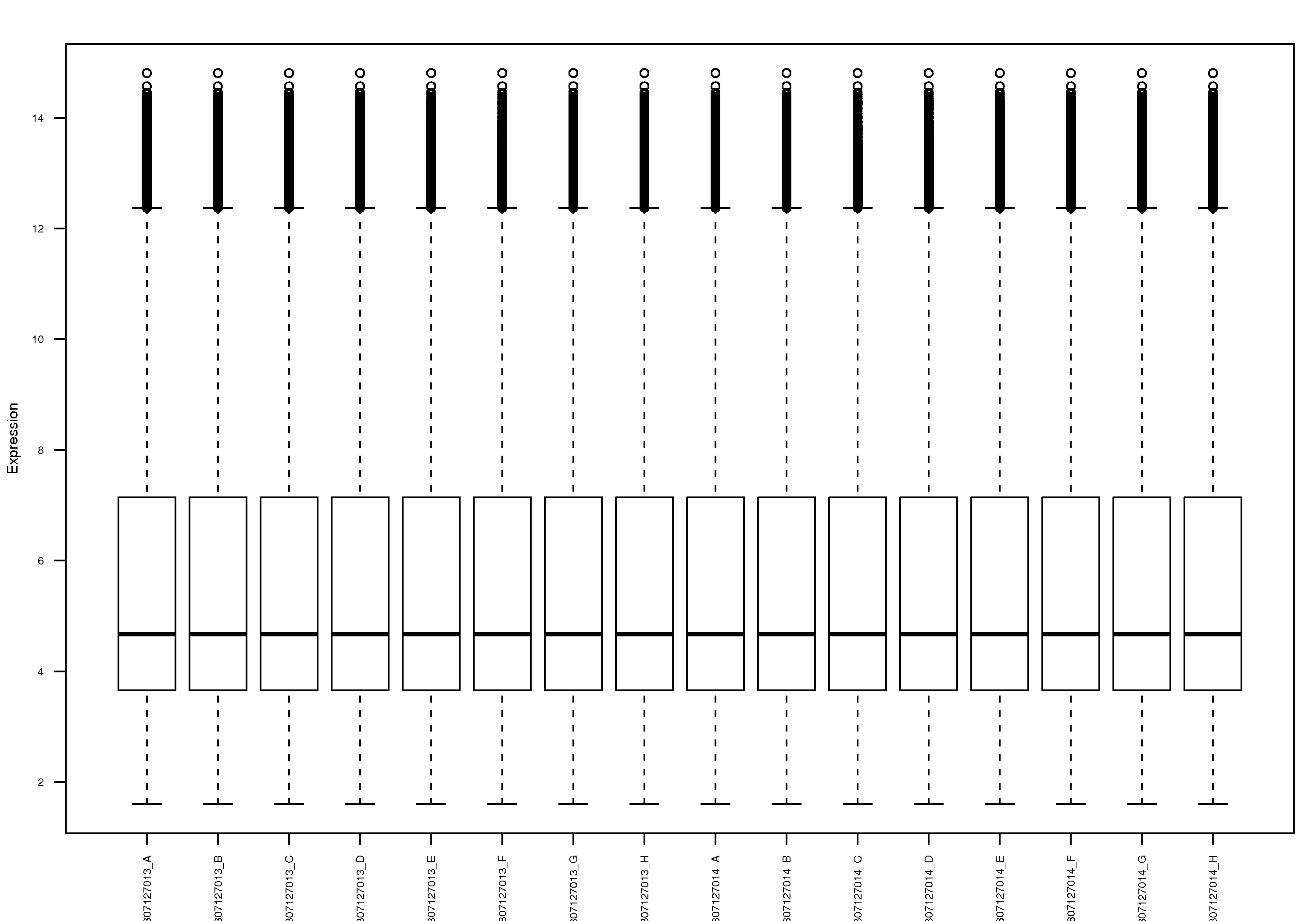


Figure SN4 Legend: Box plot of all 16 samples using post background corrected normalized intensity values. The box plots indicate that there are no anomalies or outlier samples in this dataset.

Reference:

[1] Ding, L-H., Xie, Y., Park S., Xiao G. and Story, M. D. (2008) Enhanced identification and biological validation of differential gene expression via Illumina whole-genome expression arrays through the use of the model-based background correction methodology, *Nucleic Acids Research,* 36(10) **e58** doi:10.1093/nar/gkn234

[2] Bolstad, B. M., Irizarry R. A., Astrand, M, and Speed, T. P. (2003) A Comparison of Normalization Methods for High Density Oligonucleotide Array Data Based on Bias and Variance. Bioinformatics 19(2) ,pp 185-193.

[3] R programming language: www.r-package.org

[4] Benjamini, Y., and Hochberg, Y. (1995). Controlling the false discovery rate: a practical and powerful approach to multiple testing. Journal of the Royal Statistical Society Series B, **57**, 289–300.
